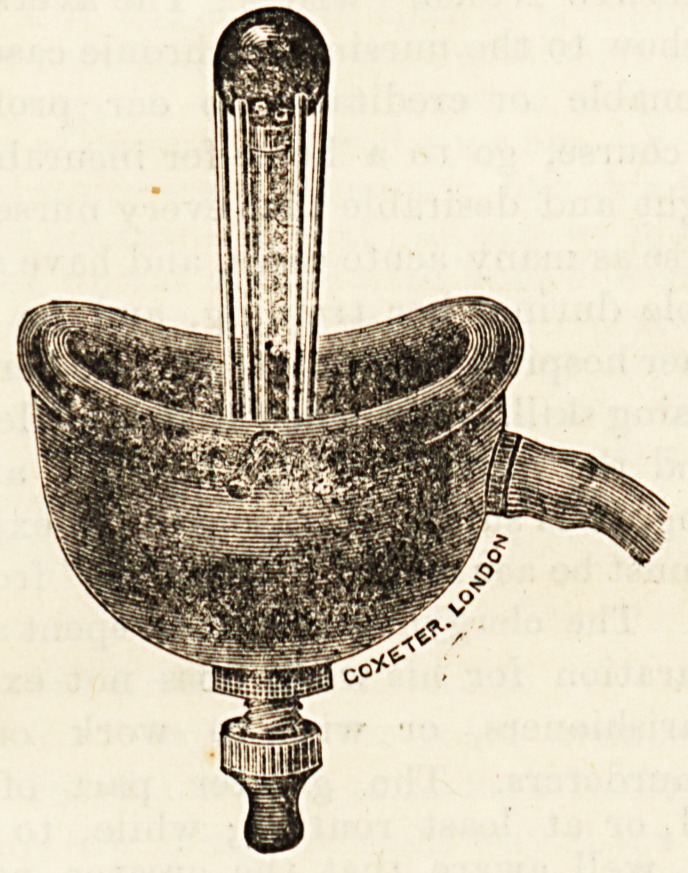# "The Hospital" Nursing Mirror

**Published:** 1900-04-07

**Authors:** 


### "The Hospital" Nursing Mirror.

Being the Nursing Section of "The Hospital."

[Contributions for this Section of "The Hospital" should be addressed to the Editor, The Hospital, 28 & 29, Southampton Street, Strand, London, W.C., and should have the word "Nursing" plainly written in left-hand top corner of the envelope.]

### Notes on News from the Nursing World.

### THE PRINCESS OF WALES'S HOSPITAL FOR WOUNDED CONVALESCENT SOLDIERS.

How deeply the Princess of Wales is interested in the wounded soldiers who are brought home from South Africa in her hospital ship was shown by the fact that on Tuesday, the eve of her departure to Denmark, she, in company with the Prince of Wales, visited the private military hospital at Surbiton, which Mr. and Mrs. Alfred Cooper have established in their grounds at The Gables. A full account of this charming and most useful institution appears in another column, together with the statements of several soldiers in regard to their treatment on the vessel, which finally disposes of Sergeant Harper's assertions. The Prince and Princess, having visited the wards, and expressed themselves highly pleased with all the arrangements, Her Royal Highness presented each of the inmates with a soldier's Testament and pocket case. The latter, which was inscribed "A gift from the Princess of Wales," contained a portrait of Her Royal Highness, a note tablet, a cartridge, pencil, a pocket-knife, and a pair of scissors.

### THE WELSH HOSPITAL.

On Tuesday the Prince of Wales inspected, at Marlborough House, the staff of the Welsh Hospital about to leave for service in South Africa. The Prince was accompanied by the Princess of Wales and Princess Victoria of Wales, and, in expressing his satisfaction with the appearance of the staff, wished them a safe journey out and a safe return home, "after carrying out their duties with credit to themselves and to Wales." Lady Parker introduced the matron, Miss Marion Lloyd, and the nurses, the Misses Pugh, Martin, Lloyd, Alice Williams, Bulkeley Williams, Owen, Jones, Lewis, and Sage.

### THE NUMBER OF NURSES IN SOUTH AFRICA.

In reply to a question by Mr. Weir in the House of Commons, on Tuesday, the Under Secretary for War said: "The total number of nurses employed in South Africa is 514 at rates of pay varying from £30 a year with allowances, in the case of the Army Nursing Service, to two guineas a week in the case of those employed locally or accompanying invalids home. About 2,000 applications for such employment were received, but no record has been kept to show how many offered their services free of charge." It will be seen by this that the supply of nurses is greatly in excess of the demand.

### MORE NURSES WANTED AT MOOI RIVER.

Sir William Thomson, referring to his visit to the Mooi River Hospital, states that at the time there were only nine nursing sisters at work, and that nursing, in the sense that it is ordinarily understood, was therefore practically out of the question. A general superintendence, Sir William points out, was really all that the sisters could provide—"valuable beyond question, still of necessity so limited that its value was reduced to the smallest compass." Sir William contends that "if the principle is recognised of having nurses in hospitals at all, a sufficient number should be provided to enable real nursing to be carried out." There seems a suggestion here that it might be better not to recognise the principle, a view to which, of course, we do not subscribe, but the importance of a staff large enough to ensure real nursing is unquestionable. We are afraid that the strange indisposition of the authorities to send out enough nurses to the front, from which they are now tardily recovering, justifies criticism like that of Sir William Thomson, which it would have been so easy and so cheap to have avoided.

### NURSING AT THE BLOEMFONTEIN HOSPITAL.

A correspondent of the *Daily News* who was taken prisoner by the Boers speaks highly of their treatment of the wounded. Referring to the Bloemfontein Hospital, he says, "Everything is as near perfection from a medical and surgical point as any sane man can hope to see. It is an extensive institution. One end is set apart for the Boer wounded, the other for the British. No difference is made between the two with regard to accommodation, food, medical attendants, nursing, or visiting. Concerning our matron, Miss M. M. Young, and nurses, all I can say is, that they are gentlewomen of the highest type, of whom any nation in the world might well be proud." This was, of course, written before the occupation of Bloemfontein by the British; but it is satisfactory to learn, on good authority, that the present nursing in that town is of a very superior character to the nursing at Johannesburg.

### ST. GEORGE'S INFIRMARY, FULHAM ROAD.—ANOTHER NEW DEPARTURE.

Although the Guardians of St. George's, Hanover Square, Union have not made, and do not seem to contemplate, any improvement of the patients' food, or any arrangement for the separation of children from adults, they have taken another new departure. They have drawn up a set of rules which it is "preferred" that the lady visitors shall sign annually. These are three in number, and the sting lies in the second, which is to the effect "that no lady visitor shall in any way interfere with the customs, or rules, or anything else of the infirmary," though "she may at any time bring to the notice of the Guardians such points as she thinks worthy of their consideration." The curious thing is that this simple and unobjectionable rule should not have been passed until one of the lady visitors reported to The Hospital "Nursing Mirror" some serious instances of mismanagement.

### THE ROYAL NATIONAL PENSION FUND FOR NURSES.

The Hon. Secretary of the Junius S. Morgan Benevolent Fund requests us to ask nurses of the Pension Fund to address their contributions to the Benevolent Fund at 28, Finsbury Pavement, E.C., together with their name and policy number. Her circular letter to policy-holders has already been responded to by upwards of 114 nurses, but as many have appeared to be uncertain as to the address to which contributions should be sent the hon. secretary thinks further notification in the "Nursing Mirror" will be useful to those who have not already contributed.

### BRIGHTON PEOPLE AND THE QUEEN'S NURSES.

IT appears that Brighton is one of the few important towns in the country where the Queen's Nurses, instead of being treated with consideration by the local authorities, are distinctly discouraged. For instance, at a meeting of the council of the Brighton and Hove and Preston Nursing Association, Mrs. Eggar mentioned that a grant of only £10 10s. was obtained with difficulty from the Board of Guardians; that of 32 parishes in Brighton only 12 made grants, and none in Hove and Preston; and that applications for the nurses to ride in the omnibuses and trains when on duty free of charge had been disregarded, though the hon. secretary had interviewed each director of the Omnibus Company; and Mr. Gerald Loder, M.P., had pleaded with the directors of the Brighton Railway. In most towns, it was urged, the Queen's Nurses are recognised as the police force nurses; but when they saved a policeman's life at Brighton and then wrote to the Watch Committee asking, not for help, but for some recognition, they received an abrupt reply. "All round, indeed," continued Mrs. Eggar, "the association, beloved as it is by the poor, seems to meet with rebuffs and lack of sympathy." This condition of affairs is not creditable to Brighton people, who can afford to give adequate support to the Nursing Association, and whose public men should be sufficiently well acquainted with the work done by the nurses to render them every assistance in their power. It is only fair to the Mayor to add that he has spoken in warm terms of the good done by the Association, and expressed a personal desire that it should be in a position to employ several more nurses.

### KENT AND CANTERBURY HOSPITAL.

WE recently received a communication complaining of the inability of the nursing staff at the Kent and Canterbury Hospital to secure much-needed reforms. It was stated that a petition had twice been presented to the Board of Management, and that practically no notice had been taken of the complaints made. On inquiry, we are informed by the Dean of Canterbury, who is chairman of the board, that all the allegations were thoroughly and exhaustively examined by a Select Committee, and that every real ground of complaint was most carefully considered and has been energetically dealt with. "At the present moment," Dean Farrar says, "everything has been put on a most excellent footing, and is going on quite satisfactorily."

### THE AGE LIMIT AT ADDENBROOKE'S HOSPITAL.

AT the quarterly court of the governors of Addenbrooke's Hospital the question of age limit was discussed.

"The Weekly Board recommended the following in the case of nurses to be hereinafter appointed: '(1) That a member of the nursing staff shall be allowed to retain her office after the age of 60 years only by permission of the Weekly Board; (2) every member of the nursing staff shall, on her appointment, produce her certificate of birth or other satisfactory evidence of her age, and the date of her birth shall be entered in a book to be kept by the matron; (3) one clear quarter before the date at which any member of the nursing staff will reach the age of 60 years the matron shall bring the case of such member of the staff before the notice of the Weekly Board.'"

Dr. Cooper and other speakers thought that some regulation should be made as to the age at which nurses should be admitted, but in the end the report was agreed to as it stood. If there is to be an age limit at all for nurses that of 60 is not unreasonable, and the pension of £40 a year which has been sanctioned in the case of two sisters who have just been invited to retire is certainly liberal. The mere fact, however, that the Weekly Board retain power to allow a nurse to remain in her office after the age of 60, shows that they do not give their sanction to the idea that at that age a nurse is of necessity past work.

### DANCING ON A LARGE SCALE.

A CORRESPONDENT, referring to our comments last week on dress at a nurses' dance, encloses us a report from a Glasgow paper of a nurses' dance on a very big scale indeed. The function took place at the City Chambers, Glasgow, and no less than 272 nurses were present. Our correspondent wishes to know "whether we object to a dance of this kind," and points out that "at least it is not open to the objection that it took place in a hospital in the vicinity of patients." This is true, but we observe that the function is called the nurses' dance; that the nurses were all dressed in new uniforms; and that the Lord Provost, speaking in the interval, advocated such entertainments for nurses. Our attitude is in accord with that of Miss Hopper, the matron of the new Bethnal Green Infirmary, who, in an interview reported elsewhere in these pages, protests against the idea that dances should be provided for nurses. By all means let nurses in their private capacity enjoy as much dancing as they can; but we really do not see why, in their professional capacity, they should have a ball got up for them either in a hospital or in a public hall, any more than the members of any other profession.

### SUFFOLK NURSING ASSOCIATION.

AT the third annual meeting of subscribers and friends of the Suffolk Nursing Association, held in the Town Hall, Ipswich, the annual report was adopted. It contains several interesting points. There is still, it seems, a difficulty in getting women to come forward to train as nurses, though all the scholarships but one were awarded last year and six nurses were trained. The demand for nurses increased year by year all over the country, and it was almost an impossibility to get a cottage nurse ready trained in return for the salaries offered, so that districts when formed had often to wait six months and longer before a nurse could be trained for them. The scheme, originated by Miss Sibyl Grant, of having a supply of nurses living in a home, fully-trained nurses for private cases, and cottage nurses for nursing a large district round Hitcham, and sending out extra nurses to districts affiliated to the Suffolk Nursing Association, will, it is hoped, be the greatest boon to the county. An effort is being made to start a sick and pension fund for the nurses' benefit. The Association has decided on an affiliation fee of not less than 5s. per annum from each district. A nurses' library was being started, Lady Constance Barne having given a donation for the purpose of buying a few medical books for the use of the nurses, and thus forming a nucleus of a library. It is intended, as soon as the requisite money and the necessary supply of nurses can be obtained, to cover the whole county with branches of the association.

### A NURSE QUALIFIES FOR THE POST OF SANITARY INSPECTOR.

At the recent examination in Birmingham of "the London Sanitary Institute" for sanitary inspectors 34 candidates presented themselves; 19 were successful, three of them being ladies, and one, Miss Kathleen Scott-O'Connor, a "Queen's nurse." It may be worth while to remind our readers that nurses are eligible to enter for these examinations. At the same time it must be stated that passing the examination and obtaining an appointment are two very different affairs.

### THE VIENNA SCANDAL.

A TRIAL for libel which has just been concluded at Vienna in connection with a private hospital for children is notable for the fact that, according to the *Times*, "painful evidence of neglect and over-rough treatment on the part both of the doctors and the nuns who acted as nurses was given during the proceedings by the parents of some of the unfortunate children entrusted to their care. Their testimony, which," our contemporary says, "abounded with details of a sensational and even dramatic character, could not be rebutted to the satisfaction of the jury." It appears that the hospital in question is overcrowded, while the medical attendance is wholly inadequate. Both doctors and nurses are harassed and overworked, and, although there can be no excuse for such a state of things as has just come to light, the *Times* is informed that the blame does not rest by any means exclusively with those who played the leading part.

### THE EAST LONDON NURSING SOCIETY.

THE annual meeting of the East London Nursing Society will be held at the Mansion House on Tuesday afternoon, April 24th, the Lord Mayor in the chair. In addition to several of the well-known clergy and laity, the matrons and nurses will be present. Another function in connection with the society will be the usual special service on Tuesday, June 12th, in St. Paul's Cathedral. The report discloses the increasingly large operations of the organisation. The number of persons nursed by the 31 nurses working among a population of 337,800 in 1899 was 5,306, as against 5,187 in 1898, and the number of visits recorded 120,022, as compared with 116,020. It is stated that the year has been a very trying one for the staff, none of whom escaped being attacked by ailments prevailing in the district. Nurse Harding, after fifteen years' devoted service, and Nurse Crees, after five, retired. Mention is made of the severe loss sustained by the death of Mrs. Stuart-Wortley, who was a warm friend of the society. We quote, with pleasure, the striking tribute paid to the memory of a truly noble woman:—

"Her influence was widely felt; our nurses knew how she cared for them, and also how high was the view she took of the work to which they have devoted their powers. Few things in our yearly course were more striking or beautiful than to see the nurses watching for her, and gathering round her, when we met in our great Cathedral for our service of prayer and praise. The great dignity of her character was united with such gracious womanhood that all honoured and respected, as much as they loved and trusted, her. Her care for others continued to the last. She never spared herself, but often came long distances, and gave up many pleasures that she might be at her post to help us, and, when failing health made that impossible, one of her last occupations was to take part in making arrangements for a village nurse at her country home."

### CENTRAL BUREAU FOR THE EMPLOYMENT OF WOMEN.

IN the second annual report issued by the Central Bureau it is stated that among the appointments effected in 1899 are the following:—Two librarians, two cookery teachers, a secretary to a factory inspector, a secretary to an editor, a rent collector, a matron of a convalescent home, a matron of an orphanage, drill teachers to the London School Board, an accountant at a girls' college, a laundry manageress, and a laundry superintendent. During the year 25 women have followed suggestions given at the Bureau for definite training, including two ladies who have become students at the House for Home-life Training, started as an experiment by the Sesame Club; and one lady who is working for a year as an apprentice with a West-end firm of house decorators.

### HOMES OF COMFORT.

LAST year the Meath Home of Comfort for Epileptic Women and Girls at Godalming experienced its first really serious epidemic. There were no less than 61 cases of influenza, which included the assistant matron, three nurses, and three servants. Every institution in London was tried in vain for extra nurses, and the heavy task of nursing all these cases devolved upon the lady superintendent, Mrs. Cousins, and the remaining nurses who managed to keep about. The need of the home for a cottage by the sea has been very much felt since the patients recovered, and it is to be hoped that the vision of possessing one at an early date will be realised this year. The work done at Godalming is of an excellent character, and we are not surprised to learn that a lady Poor Law guardian from the Midlands who visited the home has reported most favourably of it to her Board. Although partly self-supporting, the institution is in need of more voluntary contributions. Admirable work is also being done by the Children's Home Hospital at Barnet. Fifty-one cases were treated last year, of whom no less than 33 were tuberculous. In the interesting annual report it is stated that the life of the home is centred in the doctors and sister-in-charge, whose unwearied efforts for the welfare of the patients is warmly acknowledged. The 15 beds at the disposal of the committee continue to be given by preference to surgical cases requiring careful nursing, which are not eligible for ordinary convalescent homes.

### SHORT ITEMS.

A NATIONAL bazaar, under Royal and distinguished patronage in aid of the sufferers of the war, will be held at the Empress Rooms, Royal Palace Hotel, Kensington, and adjoining grounds, which have been placed at the disposal of the committee, on May 24th, 25th, and 26th. The stalls are to represent all the regiments now serving at the front.—The annual report of that excellent charity the Irish Distressed Ladies' Fund shows that expenditure exceeded the receipts by nearly £700, and that it has therefore been necessary to draw largely on the small capital in order to meet pressing cases. This year, it may be hoped, the fund will attain the increased financial support it urgently needs.—Miss Edith C. Fry, matron of Norwood Cottage Hospital, wishes to explain that she is not the Miss Edith Fry who went out to South Africa in the s.s. "Briton" on the 24th ult. She has no intention of going to South Africa at present.

### Lectures on Medicine to Nurses.

By H. A. Latimer, M.D. (Dunelm), M.R.C.S. Eng., L.S.A.Lond.; Consulting Surgeon, Swansea Hospital; Past President of the South Wales and Monmouthshire Branch of Brit. Med. Assoc.; Past President of the Swansea Medical Society; Hon. Life Member of the St. John Ambulance Association, &c.

### LECTURE V.

### Symptoms and Signs—Their Nature and Value.

When an organ is unable to perform its appropriate work easily and naturally it manifests the disturbance either by some derangement of its normal action or by pain, and such a manifestation goes by the name of a "symptom" of disease. It is a rational way of looking at the affair to consider, first of all, what the natural work of a part is, and, having done this, to see in what way a variation in this work is taking place. Any such variation is a token or symptom of some disturbance at the part affected, and calls for notice if severe enough to annoy or to give pain.

Looked at from this point of view it is easy to give you a few examples on the subject offhand. It is, for instance, the function of the lungs to aërate the blood by the acts of dilating and contracting in response to the movements of the chest walls in breathing; such acts should be performed automatically and in such an easy manner that we are not even aware that they are taking place. Should the breathing become hurried and painful we have a symptom of some disturbance of the lungs—not necessarily the result of an intrinsic disease there, but calling for an investigation of these organs as to their health. If one could say for a certainty, on finding hurried breathing, that the lungs were at fault, the practice of medicine would be one of the simplest callings, for we should then only have to examine these organs to at once find out what is the matter with them; but as the faulty breathing may arise from some affection of the nervous system, or of the heart, or of the blood, besides mischief seated in the lungs or the pleura, there is a deeper problem to be solved than would at first sight appear to be the case. All we can say at first is: Here is a symptom; let us see what it means. Take another example, the function of the stomach is to perform certain acts of digestion of food. Ordinarily we eat and go about our businesses, and, beyond a sense of repletion if we have indulged somewhat heavily in the meal, we take no further notice of what we have done. But it may be that we experience unpleasant, uneasy, or painful feelings at the seat of the stomach, or we are troubled by nausea or sickness. In such a case there is evidently some disturbance affecting the stomach, and the feeling I have spoken of, with others which I have not mentioned, become symptoms of illness. Again, a person feels ill, being troubled by a sense of lassitude, with headache, want of appetite, and feverishness; he sleeps badly, or awakes from sleep unrefreshed by the night's repose. Here we have symptoms of some general disturbance, and it behoves us to mark them and to ascertain their cause. I might multiply such examples as these were it necessary to do so; but I shall not do so, for I think I have said enough to mark what I want you to notice, viz., that by various disturbances of health discomforts and pains, called symptoms, are produced, and that these tokens of disease are of so indefinite a character that we have to make careful investigation of them to ascertain what they really arise from. Such general symptoms may be common to many diseases, and oftentimes do not in themselves possess any marked feature proclaiming them to have a special origin. The mere derangement of the quality of the blood by the presence in it of some poisonous material derived from without, or caused by faulty action of one or more of the organs whose work it is to remove such poisons from the system, will set up fever, headache, pains in the limbs, loss of appetite, and a general sense of ill-health. The acumen of the physician has to be exercised to discover the cause of such derangements, for there is nothing in themselves which tell a distinctive story of their origin. But it is quite another affair with the symptom which is called a sign, for here something of an absolutely distinctive character shows itself by which we are enabled to say that we are dealing with a certain definite disease. In choosing some examples of signs, I cannot do better than in selecting some of the specific fevers which have peculiar rashes attending them. Let me take measles, for one instance. Here a child—for it is generally in childhood that we meet with this complaint—will suffer from all the symptoms of a feverish cold which has affected the air passages. He will cough and sneeze, may have headache, and will be feverish. You see these are nothing of themselves to show that he has anything more than a feverish cold, with perhaps some bronchitis. But after some days of such illness a rash appears about the head and neck and subsequently upon the body and legs. Not only does a rash show itself, but there is a peculiarity about the appearance of the eruption, for it has an especial form which stamps it. On seeing it we say: Now there is no further obscurity about the case, for a sign pointing definitely to measles has made its appearance, and we diagnose that disease as being what the child is suffering from. Even signs are not always so definite as the one I have now adduced as an example; they may have to be read in conjunction with other symptoms to clinch a diagnosis and so establish an absolute opinion on the nature of a disease we are considering. We may, for instance, find sugar on chemically testing a specimen of urine, and yet the patient may not be suffering from diabetes mellitus, for there is such a condition as that of the transient appearance of sugar in that fluid; but if it is present in specimen after specimen of urine, and if, moreover, the patient who yields it is suffering from other symptoms of that disease, we can justly regard it as a sign. I have devoted some time to a full consideration of the subject of symptoms in their general aspect. I wish you to thoroughly grasp their significance. I may be told that such matters have nothing to do with the duties of a nurse, and that all she is required to do is to carry out the directions of the medical man who is in attendance on a case, and to see that patient's pillows are adjusted as required, that he is properly fed, that his bed is well made, and that she is at hand to help him when help is necessary. There are a good many unthinking people in the world who oppose the diffusion of knowledge, holding that it should be retained in special classes. I am not one of these, nor, I am sure, are you. As a matter of fact, the information you can give a physician as to the peculiar features of an illness, and about the advent of new symptoms in it, is of the greatest value to him. It may be that you have observed some feature of a case which is absent when he sees a patient, and that by the intelligent use of what you have seen as communicated to him, he may be enabled to form a correct diagnosis where otherwise he would fail, from having nothing to guide him. This is especially the case with regard to the specific fevers which are accompanied by rashes, for ofttimes these rashes are of the most fugitive character and have come and gone again before he appears on the scene, and he is dependent upon reports as to their having appeared at all and as to their character when they have shown themselves. Grave symptoms, helping greatly in the establishment of a diagnosis, are often fugitive; hence it is of the greatest value to a doctor to be assisted by an observant nurse. So the moral of this lecture is summed up when I tell you to be diligent in observation when attending the sick and to make a record of what you have seen for the physician's guidance. If you do this well he will be thankful for your aid; if you are also well skilled in the technique of your calling you will be an exceedingly valuable person in the sick room.

### The Only Private Military Hospital in England.

By Our Commissioner.

### PATIENTS FROM THE "PRINCESS OF WALES."

In The Hospital "Nursing Mirror" last week the allegation of Sergeant Harper that the wounded soldiers on board the "Princess of Wales" hospital ship were indifferently treated, received an emphatic and satisfactory contradiction from the military authorities. But in order to ascertain the views of the men themselves first-hand I paid a visit on Saturday to The Gables Hospital at Surbiton. Here, thanks to the generosity of Mr. and Mrs. Alfred Cooper, a number of "Tommies" from South Africa are provided with surgical and medical attendance, careful nursing, and constant recreation. The offer of Mr. and Mrs. Cooper to provide for a portion of the wounded returning in the "Princess of Wales'" hospital ship was accepted at the wish of Her Royal Highness, who was doubtless impressed by the many advantages offered. The charming residence, with its large and beautiful grounds, is so close to Surbiton Station on the main line from Netley, that any bad cases could be easily carried into the hospital. As to the building itself, it has literally been converted from a model theatre into a model hospital. The illustration conveys a fair idea of the chief ward, taken from the stage of the theatre. Underneath the motto "Nothing like Perseverance," at the end of the ward, Mrs. Cooper places fresh flowers every morning. On the floor below are two more auxiliary wards, a commodious mess-room, a well-equipped kitchen, lavatories, and a big curtained-off bath; while on the same floor as the principal ward is an operating room perfectly appointed. There are six Surbiton surgeons attached to the hospital, and Mr. Abbot, of St. Thomas's, volunteered his services for consulting purposes. Miss Spencer, who was trained at the London Hospital and has had experience at Haslar, is the matron, and a sergeant is in military charge of the men.

The Chief Ward of the Gables Hospital.

### The Number of Patients.

As Mr. Cooper, in showing me round, observed, "The Gables is not a convalescent home, but is intended to receive the wounded on their arrival from the front, however great their injuries."

"Have you had any operations?" I inquired.

"Only one," he rejoined; "but we are quite prepared for bad cases, and should like to have the gratification of treating some satisfactorily. The men remain about a month, and we intend to keep the hospital open as long as the war lasts."

"And how many beds are occupied?"

"Twenty-seven. We could, however, make room for thirty." Here Mr. Cooper introduced me to the matron, who, like the sergeant, has a room of her own close to the chief ward; and then he said, "You would like to ask the men questions?"

### Experiences on the "Princess of Wales."

Curiously enough the very first man I interrogated was Private Forbes, of the Argyll and Sutherland Highlanders, the same regiment as Sergeant Harper's, who, when I asked him how he was treated on board the "Princess of Wales," answered with a bright smile,

"Extremely well. Of the two I found it better than Netley." As he had no complaint to make of Netley this was conclusive, and I passed on with Mr. Cooper into one of the small wards where there were two patients, one of whom was receiving massage. To the other, Private Booth, of the Grenadier Guards, I put several questions.

"We got plenty to eat," he said, "and though it is true that there was no meal from half-past four in the afternoon till eight next morning, that is the rule in all military hospitals. As to smoking, Major Morgan used to serve out every week a quarter-pound of cake tobacco or 50 cigarettes to each man. The dinner was better than any I ever get in barracks, and the doctor would always put us down for anything we wanted. He came to see us daily."

"And the nurses?" I inquired.

"Miss Chadwick, the superintendent, was very good to all of us, and the three nurses were most attentive."

Next I chatted with Private Bunkell, of the Seaforth Highlanders, who was in the convalescent ward of the vessel.

"There were no complaints in my ward," he said, "and Captain Pearse often asked me if I wanted the diet changed. Though we had no meal after half-past four, we often had fruit, and in the hot climate plenty of grapes were given to us, also water-melons. Those who needed port wine or sherry got it, and total abstainers were given aerated waters."

"Can you tell me anything about Sergeant Harper?"

"He was shot in the head, and his wound may have affected him. Anyhow, he was a notorious grumbler, and was never satisfied. At Wynberg he used to grumble about his letters. As to smoking, of course we could not smoke below, but we had such a liberal supply that I brought a box of cigarettes off the ship."

### Nothing to Complain Of.

"I think," observed Private Robert Pendergast, of the Argyll and Sutherland Highlanders, "that we were treated on the 'Princess of Wales' ship just as we ought to have been. I can assure you we appreciated the difference between the treatment going out and coming back. Sergeant Harper is quite mistaken. Not only is it not true that we did not know what it was to have a square meal, but the dinner was splendid. We had soup two or three times a week, and always plenty of hot meat and vegetables. I always had enough to eat. As to smoking, I am a very heavy smoker, but I never was short of tobacco. The sister was never away from the Princess Louise ward, attending the patients and bandaging them up. Sergeant Harper was an inveterate grumbler. There was nothing whatever to complain of."

Private Philpott, of the 3rd Grenadier Guards, and Private Griffin, of the 1st Gordons, who were both in the convalescent ward of the hospital, also expressed themselves "satisfied wi h everything." Griffin, who was badly hurt in the arm at Magersfontein, tersely summed up the situation by saying "I always had enough to eat, and plenty to smoke."

Then I "interviewed" Private W. McKay, of the 1st Highland Light Infantry, who, in conjunction with Private D. McMillan, of the 2nd Seaforth Highlanders, has described the battle of Magersfontein in stirring verse.

"I was in the Princess Louise ward," he said, "along with Sergeant Harper, and I can tell you that we had plenty of meals and well-cooked food. We should have been glad if tea had been a little later, but we had all we required. If we wanted tobacco or cigarettes, we had only to ask Major Morgan, and the sister was most attentive."

### Their Present Quarters.

As I felt that I had now obtained sufficient direct personal testimony in refutation of Sergeant Harper's complaints, I asked some of the men whether they had any fault to find with their present quarters.

"Fault!" rejoined one, "why even Harper could hardly grumble here. We have anything we want, and the only cause for complaint with most of us is that we shall have to leave. We not only have ample meals, and smoking all day long if we choose, but we have all kinds of games, including bagatelle, tennis, football, and various entertainments. The men who come here are lucky indeed."

### The Regulations.

It must not be supposed that hospital discipline is not observed. My eye caught sight of the placard, "Rules and Regulations," according to which the meals are: Breakfast, quarter to eight; light lunch, half-past ten; dinner, quarter to one; tea, four; supper, seven; bedtime, nine. Patients are permitted to use all parts of the grounds; to smoke at all hours, indoors and out, up to bedtime; and visitors are allowed at reasonable times, with the approval of the matron. The final passage is: "These regulations are made for the comfort and convenience of patients, and it is therefore confidently hoped that every man will cheerfully assist in maintaining them."

"And how far," I asked Mr. and Mrs. Cooper subsequently, "has this hope been realised?"

"We have nothing but praise for the men," replied Mr. Cooper. "I thought it better to put them on their honour, and they have not disappointed us. During the whole of the time they have been here I have heard no bad language, and, in fact, their behaviour has been in all respects excellent."

Mrs. Cooper confessed that she had some misgivings before the men came, and alarmist pictures of possibilities had been painted to her. "But," she continued, "their conduct has been as good as their gratitude is genuine. They are very much touched to know that the Princess of Wales takes such an interest in them." An evidence of this was forthcoming just before I quitted The Gables, for in response to the congratulations sent by the men to the Prince of Wales on his success at the Grand National Steeplechase, a telegram of thanks was received, addressed to "the wounded soldiers at the Princess of Wales' Hospital, at Surbiton," which was read amid enthusiastic applause. Then the patients prepared to witness a shooting match in the grounds between ten of the heroes in the field and ten volunteers, which is only one of the many forms of recreation provided for them in this most unique of military hospitals.

On leaving, I could not help feeling that the treatment the men receive here cannot fail to have a refining influence on them, calculated to create a lifelong impression.

### A Changed Opinion.

When I was a pro., and sore oppressed
By the rush of a children's ward,
I gazed with longing eyes at the dress
Which charge-nurses' posts afford.
I yearned for the day when I should break
From prodom's chrysalis,
And emerge a bright and glorious "charge,"
And my hourly cry was this—
Oh, would I were a "charge"!
*She* has no macs. to scrub,
*She* has no tins to rub—
Nothing to do but pace the ground,
And watch the hard-worked pros. fly round,
And gravely chide where fault is found—
A calm, majestic "charge"!

Now that I am "charge" of a spacious ward,
'Neath my rule probationers three,
Gazing wistfully back on the past, I long
Once more "a mere pro." to be;
For of all the days of one's nursing life,
Those spent as a pro. can bring
The lightsomest heart, the easiest mind,
So *now* this is what I sing—
Oh, would I were a pro.!
*She* does not organise,
*She* does not supervise—
No pros. to train, no pros. to scold,
No worries of patients manifold,
Nothing to do but to do as she's told—
A blithe, irresponsible pro.! —L. A. B.

### District Nursing in the West of Ireland.

By a Queen's Jubilee Nurse.

One of the most important things to remember in nursing the Irish people is that they have a keen sense of humour. I have caught them more than once slyly enjoying a little fun at my expense, and by far the best way is to share it with them, even when the laugh is against oneself. One evening, feeling very cold and miserable, and wondering what on earth made me become a district nurse, I found myself struggling and stumbling over slippery stones and through mud literally ankle deep. It was raining overhead, a miserable drizzle which I found intensely depressing. I had left the car on the roadside as it could go no further, and taken my way down the little "Borreen," as it is called, leading as I thought to the village. I did not anticipate any trouble in finding the house I was looking for until it dawned upon me that it was getting dark, and that I seemed to be quite on the wrong side of the little village; then suddenly the path I had been following ended in a bog. There was nothing for it but to plod my way back again over more rocks and through more mud. I had not met a soul up to this moment, therefore I was surprised, upon turning round to retrace my steps, to see two or three figures instantly disappear from where they had been outlined against the sky, evidently watching me. I got to the cottage without further difficulty, but did not meet a creature. The village seemed absolutely deserted, not even a pig was to be seen. I knocked at the door—it was opened immediately. One glance at the demure faces of the elder, and the broadly-grinning ones of the younger inmates—there being a man, a couple of women, and some children gathered round the fire—was enough to show me that my difficulties had been seen and thoroughly relished. I need hardly say that by this time I was feeling very cross, so, putting on my very sternest manner, I demanded to be shown the patient, feeling very conscious all the time of not appearing to advantage, for I was wet and blown about by the rain and wind. I also had a horrid feeling that they rather despised me than otherwise for taking so much trouble in such weather. It was too late to do anything much for the patient that night, but by being unremitting in my attention and care of the poor old man, and being able to give him some ease and comfort before he died, I succeeded in making these people respect me at last.

### The Effect of a Laugh.

Another day I went to see a patient in a village in exactly the opposite direction to the last one, and as I was not very busy at the time, I thought by giving up my morning to it I could easily walk there and back. Upon arriving at the outskirts of the village, a woman came out to greet me and to act as guide. I felt I had been visible for a long time, and as it was my first appearance there I knew that every word and look would be commented upon. All the stories I have ever read as to illicit whisky stills and the hiding of people were brought to my mind as I looked at the little cabins nestling among the hills quite hidden away in the hollows, sometimes a thin curl of smoke being the only indication of human habitation. After passing a few cottages, or hovels, my guide turned to the right to follow a so-called path winding down the hill, which after the recent rain was neither more nor less than a small mountain torrent. I stopped in despair. I felt already that I had afforded my guide much amusement by my struggles with my cloak, my bag, and my skirts generally, as also by my way of picking my steps, while she went before me as light and as surefooted as a goat. How I envied her! I half wished myself barefooted too and able to get along like that. But when at length I fairly stopped, she stopped also, and apparently with some pity for me said, "Musha, but this is a dirty place to be bringin' yer honour."

"Dirty," I exclaimed, with, I must confess, some heat, "Why its a regular mountain torrent." "Troth, and that's true for you," she coolly answered. Then the humour of the thing struck me, and I indulged in a hearty laugh, in which she joined. From that moment she seemed a different woman, and almost immediately several women and children appeared (but where they came from I do not know, no one being visible up to then), all smiling and bidding me welcome.

### A Case of Apoplexy.

It was a sad case I had been called to see. A poor woman had been seized with a fit of apoplexy about four days before; one side was quite paralysed, and she was apparently unconscious. She was lying on the floor on some straw covered with sacks, the upper part of this extemporised bed was covered with a nice clean sheet of home manufacture. Her skirts had been removed, but the jackets and bodices she was wearing when struck down were still on her, and rucked up very uncomfortably under the poor shoulders. There were about ten persons in the room, which was about fourteen feet long and twelve wide. At one end there was a huge turf fire on an open hearth, with the smoke ascending straight up through a hole in the roof, a kind of structure of sticks and plaster, not unlike an inverted funnel, guiding it out. All the inmates rose at my entrance; I inquired if the sick woman had any near relation among them; one girl stepped forward and said that she was her daughter. I kept one other girl in the room in case I needed assistance, and I then set about examining my patient. I found she had not been moved in the slightest degree since the fit, but after a couple of days the priest had been sent for. He bid them moisten her lips occasionally and send for me. Nothing else had been done for the patient. Now although this sounds very heartless they did not think so, but imagined that they were doing the very best thing to give peace to the poor sufferer. Although quite helpless and paralysed, I found she was not altogether unconscious of things passing around her. So she had the additional agony of knowing that her friends would have done anything to help her if she could have spoken and told them to lift or move her into another position from time to time. Her daughter was very anxious about her, and when upon my turning the poor mother gently over, thus, as I expected, disclosing a bedsore about as large as my hand, I explained to her how it could have been avoided, her distress became very bitter indeed. I made up the poor bed as well as I could, and arranged the sheet as a draw-sheet, showing the two girls at the same time how by its use they might turn the patient from side to side easily without disturbing her much, and so making her last hours at least more comfortable. Before leaving I prepared the yolk of an egg with a little warm milk, and got the patient to swallow about six teaspoonfuls of it. She then, with an effort, murmured the one word, "better." The daughter threw herself on to her knees beside her mother, kissing her, and saying she would suffer the pain herself rather than cause her any more, and calling her all sorts of endearing names in Irish. I only paid the patient two more visits, as she died on the third day.

### The Characteristics of the People.

On the whole I find these poor people most courteous and obliging, thoroughly grateful for the least thing done for them, especially for a kind word spoken. They certainly show more innate refinement and consideration for the feelings of others than many more cultured individuals. They never betray undue curiosity about anything, or make unpleasant personal remarks.

But it is nearly impossible to get a straight answer from them, so I seldom now put a leading question, which they seem to resent, but just, as it were, play around the subject and gather what information I want. This is the sort of conversation which goes on. A woman, complaining of a pain in her side, sent for me. She was lying on the floor beside the fire, with only the very scantiest covering. Asked how long she was bad: "Och, but it's me that's bad this long time." I gathered after a time that she had been ailing off and on for about two or three years.

"Have you a husband?"

"I have, but shure he's not much good to me."

"What does he do? Has he any work?"

"Sorra much he does, then, for he's bad himself this long time."

"Is he laid up?"

"Shure he's not, but he might as well be for all the good he does."

"In what way does he complain?"

"Musha! he doesn't complain at all, but he just has a bad cough and a cowld on his chest, and the rheumatism in all his bones this many a day."

"Well, if he doesn't work, how do you all live?" She had ten children.

"Troth, we just live the best way we can. There's a wheneen o' pitaties that himself digs up when he's able, and the sup o' milk from the cratureen of a cow, and, faith, we haven't that same itsel' now, as she's dry this couple of months."

I felt it would be only mockery to tell the poor creature to get some nourishment for herself, though I was convinced she was suffering from nothing else but pure weakness, from the want of proper sustenance. In many cases my experience is the same—want of nourishing and suitable food during their attacks of illness leaves them in such a low condition that their recovery with impaired constitution is very slow indeed. Thus the ruder constitutions survive while the weaker succumb. From one thing at least they need not suffer, namely, the want of fuel. If the land is poor in all else, it is at least rich in turf-bog, so that if the cupboard is bare at least the hearth is full of odourous, pleasant-smelling, and antiseptic peat.

Anyone dealing with these people must have a large stock of both patience and sympathy to enter into their troubles and to combat their superstitions. If, however, they once put faith in you, it will require a good deal to shake it. Several have tramped patiently and uncomplainingly for miles (Irish miles, too) for the mere purpose of seeing me and asking for a "cure." They much prefer coming to see me, and think it very strange I should go to see them. The majority of them hate the very idea of going into hospital or applying for relief. Their wants are few, and being devoid of many comforts they do not miss them. They are generous to a fault, and I have sometimes to use all my tact to avoid giving offence in refusing milk or a "small taste of the crature" (potheen) to "warm" me. On the occasion of my first or second visit some member of the family always comes out to welcome me and show me the way, and the same when leaving, thus literally fulfilling the saying, "Welcome the coming, speed the parting guest."

WORKING WITH THE DOCTOR.

In nearly all cases of serious illness, the priest is the first to be sent for, so I hear of a good many cases through him. The doctor also sends me to several from time to time. I had some difficulty at first in inducing the patients to go to the doctor after I had seen them. They considered my attendance quite sufficient, and their belief in the efficacy of medicine not being very strong, they were as a rule resigned to their fate. This made me very nervous, and in several cases where I knew it was imperative for the doctor to see them, I had to be very firm and threaten to leave them altogether. Once or twice I actually did this for a day or two. I was dreadfully afraid of the doctor at first, fearing he might think I wished to overstep my duty as a nurse, but upon becoming better acquainted with him, I found him most agreeable and kind, and only too willing to help me in every way, and to have some of the lesser troubles taken from his already overburdened shoulders. Many nurses will agree with me that it is no small pleasure to feel sure of the doctor's sympathy and help, especially in a very large district where the nurse often has to use her own judgment (there being no time to consult him) and do little things which she would never dream of doing in hospital or private nursing without first obtaining the doctor's permission. In summing up, I feel that a nurse can, if she sets about it in the right way, taking the rough with the smooth, make herself very happy in an Irish district; she will get to love her people and feel she is a power with them, and if she is a lover of nature, she will never be tired of the beautiful scenery continually surrounding her, be the season winter or summer, especially in this delightful region of Connemara.

### Practical Hints for Nurses.

### THE VALUE OF DRIPPING.

By a MATRON.

I HAVE been wondering why many of the large hospitals do not use up some of the good dripping for the patients. It would make a considerable difference in the bills for butter, as much of the latter is usually consumed. I have found myself that most patients prefer dripping when they can get it, and I think many patients on solid food would be allowed to have it by their doctors, surely approving of the pure fat from both beef and mutton. What becomes of the enormous amount of dripping there must be in most hospitals? I know the customs of many institutions, but have not yet heard of the dripping being used for the patients. We are most glad of it here, and use all dripping up; the patients gladly take it in preference to butter, both for breakfast and tea.

### BRAN CUSHIONS.

By a NURSE.

BED-SORES and their prevention must always be a subject of interest to nurses. Perhaps not all are aware how useful small bran cushions are for the prevention of pressure on certain parts, especially on the hips. I learnt this first from personal experience. My present patient is an old lady who has been bed-ridden for several months, and in spite of a water pillow and every care in the way of washing and rubbing the parts where bed-sores were likely to occur, she often felt very tender, and could not lie comfortably in any position. At last I remembered that when, as a child, I had typhoid fever abroad and complained of tenderness, my nurse brought me a bran pillow and placed it under my hip, and I felt instant relief. So I made a similar one for my patient, with most beneficial results. After using it the first night she said she had not rested so comfortably for many weeks, and I don't think she would at all like to be without it now. I think they are most useful when made rather large. The cover should be of old linen, about 12 in. long and 8 in. broad. They are best only half filled, as then you can push as much bran as you want into the required place without making the cushion too hard. Besides the convenience of them being very cheap to make, so that they can easily be renewed if they get damp or soiled, they have the additional advantages of keeping delightfully cool, and they never get hard and lumpy, as is the case with cushions stuffed with wool or tow.

### The Nurses of the New Bethnal Green Infirmary.

### A CHAT WITH THE MATRON.

By Our Commissioner.

The most perfectly-appointed infirmary in the metropolis, which, so far as the interior is concerned, may be compared to the leading general hospitals, is about to open its doors for the reception of patients. When I went to Bethnal Green the other day to have a chat with Miss Hopper, the first matron of this fine institution, the furnishers were still in possession. But the nurses' commodious and comfortable quarters were practically prepared for them, and thanks to the courtesy of the matron, I was able to inspect them.

"Of course, you have no staff yet," I said.

"At present there is no one here but the assistant matron and myself. I hope to have about eighty nurses. The number will vary a little because the wards are not all the same size. There are from thirty-eight to fifty beds on each floor. As a rule there will be a sister and two or three nurses on duty during the day, and one or two during the night, the number necessarily depending somewhat on the urgency of the cases."

"How many sisters will there be?"

"Fifteen, in addition to the matron and assistant matron. There will also be three superintendent nurses, one of whom will act as home sister."

"Your accommodation of the nursing staff seems very complete?"

"Yes, I think it is very satisfactory. As you have seen, there is a large general recreation-room, a spacious mess-room, a sitting-room for the assistant matron and superintendent nurses, a special sitting-room for the sisters, and practically separate bed-rooms for every nurse. The room for the night nurses are on separate floors, and are arranged with the view to quiet during the day.

### On New Lines.

"I presume that everything here is on new lines?"

"Yes, everything here will be run on new lines and substantially with a new staff."

"Can you tell me anything about the regulations?"

"As these are still under consideration, I cannot very well discuss them. The hours of duty are rather less than the 'London' at present, though, generally speaking, the nurses will do very well with respect to off time. They will have two hours every day, a day off once a month, and I hope we shall secure a reasonable holiday for all of them. At any rate, we hope to improve on the old-fashioned fortnight, which, when one considers the atmosphere in which most of the nurses spend their time, is decidedly too little."

### Recreation and Dancing.

"Would you like to say anything on the subject of recreation?"

"We hope to have a piano, and there is a bookcase with plenty of shelves. We have not yet begun on books. When we get the staff we shall doubtless make a start."

"May I ask your views on dancing in hospitals?"

"I think I hold the usual matron's view. I have tried the effect of dances and I have done without them, and, on the whole, the balance is against them. I have not the slightest objection to nurses dancing outside, but sooner than have dancing inside, I would prefer to give them a little extra off duty at Christmas. My conviction is that it is not a good plan for nurses to introduce what should be social entertainments into their professional life. By all means let them have everything necessary for regular recreation, such as literature and cycling, but it is a mistake for them to entertain their friends in the hospital."

"Have you any ground attached to the infirmary?"

"Only small airing grounds between the pavilions, but some parts of the roof on the administration part are flat."

### The Staff.

"Have you had a large number of applications for posts?",

"There have been over three hundred for probationerships, and a large number for superintendents and sisters."

"You will have no difficulty in forming your staff."

"None at all, so far as I can see; as a rule, there is no difficulty in obtaining sisters and probationers for a large new infirmary.

### The Matron's Career.

"In becoming matron here, you are only returning to the capital?"

"Yes, I was trained at the London Hospital, and so was Miss Minter, the assistant matron. It is eleven years since I began to train, and from the London I went to the Taunton and Somerset Hospital as sister. My next appointment was matron at Sidmouth Cottage Hospital, and from there I proceeded to Leeds as matron of the infirmary."

"That was a very great change?"

"It was a change from the charge of six to the charge of six hundred. I went to Leeds as first trained matron to begin the training school. The infirmary there is entirely separated from the workhouse. The mischief arises when they are not separated, but I am afraid this will be a burning question for a long time, because the separation involves such an enormous expense. In establishing the training school at Leeds I experienced only the ordinary difficulties, and I expect that it will be about the same here. The fact that everything is new is an attraction to many, but the other aspect of the case is that it takes some time for the staff to settle down."

### Examinations.

"I suppose the probationers will be admitted on the usual conditions?"

"They will have to pass a thorough practical and theoretical examination, and I hope we shall soon have an outside examiner. No certificate will be granted for less than three years' training, and each probationer will come for three months on trial."

"What is the nature of the medical examination?"

"The medical superintendent will examine each probationer before she is passed for the full training, and I do not consider that any other is necessary."

### Workhouse Nursing.

"I attach the greatest possible importance," continued Miss Hopper, "to the treatment of the staff, their comfort, their recreation, and their food. They should have a good and varied diet, a point that is often overlooked in the case of nurses, but it is much better than it used to be, like everything else in the nursing world. I believe that workhouse nursing is only in its infancy, and that in the future the elevation of the profession will be followed by the elevation of individual institutions. My experience of five years at Leeds has satisfied me that the wish of Guardians generally is to improve both the nursing in the infirmaries and the conditions of the patients under their care. So many nurses are now being trained that it ought soon to be easy to meet all reasonable requirements."

### Tending a Shipwrecked Crew.

By a Nurse.

The life of a nurse is often full of many and varied experiences, but the most unique which ever fell to my lot occurred a few weeks ago at Swanage, where I was spending a week or two before going to a fresh case. The weather was terrible, so wet and windy that I must confess I felt very rebellious at the prospect of having to spend my hardly-earned holiday in the house, with little to break the monotony of each day. Half of the first week had already passed, and I was beginning to wish myself "in harness" again, when one morning news reached us that a ship had run aground on the Peveril Ledge, a dangerous reef of rocks. I had never seen a wreck, so quickly donning my uniform I set out for the spot. There was a fearful south-westerly gale, and it was quite a battle to get along; time after time I was blown back and almost carried off my feet. At last I managed to reach the Peveril Point, at whose base stretched the reef of cruel rocks upon which the vessel was stranded. Never shall I forget the sight! She was so near that the crew could be seen distinctly by the crowds of people on the cliff, and yet she was in such a dangerous position that the lifeboat, which had been speedily launched, was utterly powerless to render any assistance. As the repeated efforts of the crew to reach her were unavailing, the rocket apparatus was got out on shore, and after several ineffectual attempts, the line was eventually thrown on board, but owing to its having been fastened too low down on the vessel it would not work, as it became entangled in the rocks.

The Cases.

It was a dreadful time of suspense, for it seemed hardly possible for the creaking timbers to hold together much longer. It is in such moments as these that we feel our utter helplessness. At last one of the coastguards nobly volunteered at the risk of his life to go out on the rocks and clear the line, which he did, with the waves dashing over him. Then amid great cheering the cage was sent out, and the first man brought ashore. Poor fellow, he was a fair, blue-eyed Icelander, and looked so pathetic that my nurse's heart was touched at once. He was taken to the chief coast-guard officer's house, where Dr. Cæsar Hawkins was quickly in attendance, and we soon had him rubbed and wrapped in blankets by the fire, drinking hot, strong soup. The doctor and I were just taking a little breathing space—it is no joke undressing men whose clothes are heavy with water, and undoing straps and buckles, to say nothing of removing sea boots—when another poor fellow was brought in, a Norwegian this time, the ship's cook. Then followed the captain's wife, who was more trouble than the men, being far less docile, and screaming all the time for "Carl" (her husband). She would not be parted from the money, &c., which she had sewn up in her pocket. However, we left her bewailing her husband so as to attend to a fresh case, the mate of the ship, also a Norwegian. The poor fellow had a fractured clavicle, and was very badly cut about the face. After the primary treatment he was sent to the Swanage Cottage Hospital. Our next patient was the captain himself, then a Portuguese lad, who, though only eighteen, I was informed by Hans (the cook) was already married, with one child.

A Narrow Escape.

There now remained but one man on board, Otto Von Appen, a German, but the line had got again entangled in the rocks, and the cage would not work. Everything was done to right it, but without avail, and the sailor was seen doing his best to help. At last he threw up his arms and leaped into the sea, first tying a cord round his waist, and was quickly pulled ashore, utterly exhausted and only just alive. We had a "lively" time getting him round, and then I went home to change my clothes, which were drenched, and thick with mud. I must not omit to say that I had changed my bonnet for a Tam-o'-Shanter, tied on with a scarf which was lent me. In consequence of this strange head-gear my appearance was—well, not professional. I then went back to the coastguard station and spent the night, as the last man rescued was very ill. The next morning, by the doctor's orders, I removed him to the hospital, where, after "double pneumonia," he pulled up splendidly.

The day before my departure from Swanage I had tea and spent the evening with the crew, whose gratitude was very great. They say they will never forget the kindness they received, and I am sure I shall never forget seeing my first shipwreck, or the tending of a shipwrecked crew.

### Presentations.

St. Saviour's Infirmary, East Dulwich.—Miss Florence Wooll, upon resigning the post of sister at St. Saviour's Infirmary, East Dulwich, to take up that of night superintendent at the Temperance Hospital, Hampstead, was presented with a silver teapot by the nurses with whom she had worked. Miss Wooll carries with her every good wish for success in her new post.

West Ham Hospital.—Miss Ethel Willmott, who is leaving West Ham Hospital to be married, after filling the post of matron for seven years, has been presented with a leather dressing-case, beautifully fitted, as a token of the love and esteem in which she has been held by the whole nursing staff. Dr. Grogono, senior visiting surgeon, in a few able words, made the presentation on behalf of the doctors, nurses, and servants, wishing her every happiness in her new sphere, and expressing the universal feeling of regret that she is leaving the hospital in which she has worked so long.

Gray's Hospital, Elgin.—Miss Paterson having lately resigned her appointment as matron of Gray's Hospital, and being about to leave Elgin, was waited upon at her residence, Rose Avenue, by a deputation consisting of Dr. Galletly and Dr. Adam, Mr. R. Kemp, and Lord Provost Young, and presented with a purse of sovereigns in recognition of her valuable services to the community during a period of nearly ten years. The presentation was made by Dr. Adam, who explained that the purse had been subscribed by friends of Miss Paterson who were alive to the good work she had done in Elgin. He said that Miss Paterson's matronship had been a great success; that she had brought a quite unusual degree of energy, zeal, and knowledge to bear on the great variety of duties entrusted to her, with the result that the hospital, under her management, had been a model of cleanliness and good order, and the highest degree of comfort had been secured to the patients and other inmates. He was able to speak from particular knowledge of Miss Paterson as a nurse and a trainer of nurses, and he could not speak too highly. He felt that his own efforts for the treatment of the sick during the many years he had been associated with her at Gray's Hospital could not have been more ably seconded. Miss Paterson has also received a handsome writing desk from the household staff.

### A Remedy for Sleeplessness.

By a Nurse in a Children's Hospital.

A remedy for sleeplessness which I have sometimes found useful is to fan the patient's face gently. It seems to be especially successful when the sleeplessness arises from neuralgic toothache, or when the brain is worried and the patient cannot sleep for thinking. The fanning should be done from the side, and not too close to the face. If possible the nurse should sit down, so that the patient may not be fidgetted, but have a complete sensation of rest.

### The Nurses of the Indian Army.

By an Officer of the Royal Army Medical Corps.

"In nothing do men so nearly approach the gods as in giving health to men."

I have received so many letters from nurses in my old London hospital, and lady friends elsewhere, asking the conditions under which ladies may enter the Indian Army Nursing Service that I think a brief *résumé* of the regulations governing admission to this important branch of the nursing profession may not prove uninteresting to readers of the "Hospital Nursing Mirror."

The I.A.N.S. has been organised with a view to providing lady nurses for the sick soldier and his family in India. It is an integral part of the military medical service, and consists of two grades, namely, lady superintendent and nursing sister.

### Conditions of Admission.

A candidate for the appointment of nursing sister must comply with the following five rules: She must not be under twenty-five or over thirty-five years of age, and must submit a certificate of birth to support her statement regarding her age; she must produce a certificate from some lady in society to the effect that the candidate is a fit and in every way desirable person to enter a service composed of ladies of good social position with whom she will associate, and that she possesses the tact, temper, and ability qualifying her for the appointment; she must sign a declaration on a form to be obtained from the India Office, and must have undergone three years' preliminary training and service combined in a hospital or hospitals in which adult male patients receive medical and surgical treatment, and in which a staff of nursing sisters is maintained; she must produce a certificate of health from a competent medical practitioner certifying that she is fit for service in India, certificates of efficiency in medical and surgical nursing from physicians and surgeons under whom she has served, and a letter of recommendation from the matron of the hospital in which she was trained must also be procured; she must clearly state what experience she has had in hospital supervision (as apart from nursing), and what she understands of the duties of lady superintendent of a hospital.

Having made out her application, our embryo sister should attach the necessary certificates to it and submit it to the Military Secretary, Medical Division, India Office, who will in due course acknowledge receipt.

### Term of Engagements.

The duration of term of service of a sister of the I.A.N.S. is five years; on the termination of this period she may re-engage for a further period of five years, and will be granted one year's leave to England between the two terms of service. On completion of her second five years in India, or her eleventh year of total service, a sister is granted a further year's furlough to England on two-thirds pay.

On the completion of her leave a sister may extend her service for a third period of five years. Before doing so she must be pronounced physically fit by a medical board, and be specially recommended by the Commander-in-Chief in India. In a few cases ladies have been permitted to serve a fourth period, but twenty years' service in India is too much for the health of the average Englishwoman.

The engagement between the nursing sister and the Government of India may be terminated at any time by six months' notice on either side. If a sister resigns except on the score of ill-health she must refund the sum of £20 in respect of her passage out.

Should a nurse, as sometimes happens, find it necessary to resign with less than six months' notice, she forfeits an additional £10, or £30 in all. A sister who resigns with less than two years' service is fined 100 rupees (£6 13s. 4d. sterling) in respect of her outfit allowance.

### The Duties.

With regard to duties, the members of the I.A.N.S. are under the immediate supervision and control of the superintendent, whose authority over them is supreme. The lady superintendent of each command, or province, visits every hospital in her district at least once a year, and reports confidentially to the Principal Medical Officer, her Majesty's Forces in India, on each sister individually.

Sisters are only employed in the larger stations, and have always charge of the special wards. They are assisted in their duties by orderlies supplied by the various corps stationed in the garrison, as the rank and file of the Royal Army Medical Corps does not serve in India. Their duties consist chiefly in the supervision of these orderlies and the administration of medicines, &c. They have little or no individual nursing to do except in the case of officers. Not the least important of their responsibilities is to instruct their orderlies in nursing duties, a duty which I, as a medical officer, can vouch is never shirked. Some of the regimental nursing orderlies I have had under my command in India are the best male nurses I have ever met. The sisters take turns for being on duty all day, and wherever they serve there is always one on duty till seven or eight o'clock p.m., summer and winter. If there are bad cases, one is detailed for night duty.

### The Remuneration.

Turning to the question of pay, it must be admitted that the members of the I.A.N.S. are well paid. Lady superintendents receive Rs.300 per month, or £240 sterling per annum. Sisters receive Rs.175 a month, or £140 per annum. In addition they are provided with good furnished quarters and a portion of the large number of native servants required in an Indian household. Sisters who are appointed to officiate as lady superintendent receive Rs.25 (nearly £2) per mensem extra. If sent on duty out of her own station a sister receives Rs.5 (6s. 8d.) a day deputation or travelling allowance, and if sent on field service nursing sisters get an allowance of Rs.30 a month to enable them to keep a pony. On appointment sisters receive £15 sterling to provide themselves with a suitable outfit. Lady superintendents get £25 for the same purpose.

### Pensions.

With regard to gratuities and pensions, the I.A.N.S. is fairly well off, for on completion of five years' service sisters receive a gratuity of Rs.500, with an additional Rs.140 for each year she may have been employed as lady superintendent. On completion of ten years' service a gratuity of Rs.1,500 (£100) is paid, plus Rs.250 for each year in which the recipient has acted as lady superintendent. If compelled to resign on the score of ill-health with less than five years' service, Rs.75 is granted for each year of total service, plus Rs. 200 for each year as a lady superintendent. Should a sister's health fail during her second tour of service, she is allowed Rs.175 for each year of service, and an additional Rs.240 for each year as a lady superintendent. No gratuity whatever is allowed if a sister terminates her engagement for any other cause than ill-health. On completion of 15 years' service a sister can retire on a pension of £50 per annum, with an additional £2 for every year spent in the higher grade of lady superintendent. After 20 years' service a pension of £60 is paid with the same addition, so that as a nurse's third tour is almost invariably in the higher grade, while her fourth is absolutely certain to be, a member of the Service's pension is never less than from £60 to £80 per annum, which, as pensions go, is distinctly good.

THE QUESTION OF LEAVE.

In the important matter of leave the I.A.N.S. are on exactly the same footing as officers of the British and Indian Army. Three days' temporary leave is always obtainable, and 60 days' privilege leave on full pay is granted yearly. Should a nurse by any chance fail to obtain her leave for 33 months, she is entitled to 90 days' leave on full pay. Sick leave up to six months is given in each tour if necessary, and leave out of India without pay can generally be obtained in the case of a family bereavement or similar circumstances. A free first-class passage to and from England and to and from the port of embarkation is also given.

THE SOCIAL ADVANTAGES.

Socially speaking, the members of the Indian Army Nursing Service have a very good time. They are all ladies of good social position, and, as a matter of course, mix in the best society in stations in which they may be quartered. They are usually on the best of terms with the officers of the Royal Army Medical Corps, and strained relationships between the medical and nursing staffs in Indian station hospitals are unheard of. Their pay goes very much further in India than it would go at home, and they all keep ponies and traps and indulge in games enthusiastically. There are no restrictions with regard to their attending dances and other functions.

THEIR WORK.

With respect to the good work they do among sick soldiers and officers, I cannot speak too highly, and the only pity is that there are not more of them. I should like to see their services utilised in station family hospitals instead of the half-trained midwives we have at present; but as the supervision of these hospitals is laid down in regulations as part of their duty no doubt this will come in time. For a lady who is strong enough to stand life in the tropics I know of no nobler or pleasanter professional career than that of "a nurse of the Indian Army."

### Private Monthly Nursing.

### HINTS TO THE INEXPERIENCED MONTHLY NURSE.

By A SISTER.

UNLESS a nurse is going to nurse a lady in a large, old-fashioned house, she will find it wiser not to use the much-talked-about sanitary sheet. Kitchen grates of the present day are not made big enough to burn quickly a sanitary sheet, even when it has been, with difficulty, cut up into four pieces; the consequence is that the sheet smoulders for some time, filling the kitchen and passages with an unpleasant smell, and nearly putting the fire out. An old under-blanket is far preferable to the sanitary sheet; if much soiled it can be rinsed out in the bath before being sent to the laundress; if not much soiled it can be sent as it is, with all the other things soiled at the same time. A nurse should ask her patient to make arrangements with her laundress to send the day of the confinement for all soiled linen, as, no matter how far away from the bedroom the soiled clothes may be sent, it is not right that they should remain in the house one hour longer than absolutely necessary.

MEDICAL ILLUSTRATED MIDWIFERY BOOKS.

No nurse should take a midwifery book to a patient's house. Anything she may want to know let her ask the doctor about. Only a few months ago a very young mother told me how she had dreaded her confinement. The nurse had been recommended to her by a well-known doctor, and this nurse had amused herself during her week of waiting in showing the young patient illustrations in a midwifery book. This is an unpardonable act, and ought to have been reported to the doctor who recommended her, who would most probably have crossed her name off his books. Again, some nurses are too fond of talking about "the last case I as at." What happened at "the last case" is private, and ught to be looked upon as such.

WASHING OF PATIENT.

The ordinary washhand basin is usually too big and heavy to be placed on the bed. A nurse will therefore find it to her advantage to ask her patient to buy an enamelled basin, which can be obtained for about 1s. 3d. It is light to carry, does not weigh the bedclothes down, cannot get "chipped," and is easily kept clean. It is also most useful for putting the perchloride of mercury, 1·1000, into, which is required for the disinfectant of the nurse's hands.

THE BED.

Most ladies do not change their bedroom, therefore a nurse will find her patient has either a double bed or twin bedsteads. The latter is very useful in illness, as one bed can be sed for the day and the other bed for night. If a lady has a double bed a nurse should suggest that one side be used for the day, the other side for the night. The patient then has a cool, fresh side for resting or sleeping upon.

THE BABY.

Bath the baby each morning about half an hour before his or her feeding time. If a "bottle" baby, prepare the bottle before giving the bath. If fed by the mother, sponge the breasts, so that when baby has been bathed she can be fed immediately.

THE UMBILICAL CORD.

Should the linen have stuck to the cord a nurse must not attempt to pull it off, as the warm bath will soon loosen it. Before applying a fresh piece of linen well powder the cord. The cord ought to separate at about the fourth day, but often it does not until the seventh or eighth. When it has separated put plenty of powder on the navel, then cut and round the corners of a thin post card, cover with lint (boracic in preference), place this over the navel, and then firmly put on the binder, slanting it where it has to be fastened, so that it may be tight over the umbilicus and loose over the chest. Of course, change the lint and binder each day. If the card is kept on for about ten days, the navel will be quite flat.

BABY IN BASSINETTE.

Don't place a baby between sheets, unless they are Dr. Jaeger's. Take care that the pillow is flat, that the corners are well pushed down, and that baby is not lying crooked, or with an ear "turned up." Tuck her tightly in, and put her arms under the blankets. If the baby cries don't take her out of the cot, but turn her on to her other side; often change of position is all she requires. Should this not quiet her, find out whether she wants to be made comfortable, or whether she has her legs drawn up, a sure sign that she is in pain. If she is in pain often a hot blanket placed over her legs will ease her.

CHANGING OF NAPKINS.

In hospital, nurses are taught to wash the buttocks each time of changing a baby. This, I feel, is a mistake, and is, I am sure, often the cause of sore buttocks, as the delicate skin is too tender before the month for much washing with soap and water. Nurses will find it much better to wipe off all moisture with a dry, warm towel, and then to smear the buttocks with boracic and zinc ointments or Vinolia cream. If the baby is suffering from diarrhœa or "green motions," cleanse the buttocks with soft rag or wool dipped in olive oil. A baby should never be allowed to remain "wet," no matter if nurse has "just changed her," or if "it will be feeding time in five minutes." A good nurse—a careful nurse—will change a baby frequently, if she is awake and requires attention.

### Echoes from the Outside World.

AN OPEN LETTER TO A HOSPITAL NURSE.

It is good to be able to read the headings of the newspaper, "The Queen in Ireland," and to feel that nothing has happened to prevent Her Majesty carrying out her determination to visit the Emerald Isle. One cannot help smiling at the poor folks in Kingstown making their preparations to receive their Sovereign in style, and hoping against hope that the weather might clear up, and then suddenly awaking to the fact that, not only was there no doubt of the Queen's coming, but that the Royal yacht lay quietly at rest in their very midst! The warnings of the barometer had been so emphatic that her Majesty had been obliged to upset her official programme, and arrive three or four hours before she was expected. She has expressed her sorrow that her coming should have taken the form of a "surprise" visit, because she knew the disappointment that it must have inflicted on her loyal Irish subjects, but if she had not crossed when she could she might have been delayed some time, which would have been disastrous to all the arrangements. Strangely enough, when George IV. reached the shores of Ireland he was equally unlooked for, and was only received at Howth by a small group of farmers and fishermen. So history repeats itself. The Queen's reception at Dublin on Wednesday was magnificent, and even the weather behaved itself splendidly.

The report of the death of General Joubert, to which I alluded last week, proved to be well-founded. The commander whom Sir George White spoke of as "a soldier and a gentleman, and a brave and honourable opponent" died on Tuesday at Pretoria of peritonitis, his last words being "My poor people! My poor country! What will become of it?" By his special desire General Louis Botha is to succeed him as Commandant-General of the Boer forces. I am told by a friend from South Africa that at the commencement of the war the Colonials were more afraid of General Botha than of anyone, because he had lived in Natal for many years, had farmed at Greytown, and knew the country by heart. His two sisters are Loyalists, one being married to a Durban doctor, and they naturally deeply deplore the present state of affairs. As to our own position, we are waiting for news of a big battle which seems impending near Bloemfontein. The Boers appear to have rallied a little from the effect of their late disasters. Commandant Olivier having, by a clever piece of strategy, taken advantage of the only vulnerable point in Lord Roberts' position, and retaken Thaba Nchu. The waterworks of the Free State capital have been seized, which is not as serious as it might be because Bloemfontein has other means of getting water, but the farmers who have surrendered to us are likely to be hardly dealt with by the enemy. The loss of a convoy and five guns under charge of Colonel Broadwood is a disaster, but it is a matter of congratulation that any of the men or the guns escaped from the trap into which they fell, and the gallantry of our force was so marked that even the Boers were constrained to admire.

An interesting letter reaches me from one of the Natal Carbineers who entered Ladysmith with the relieving column. To him the occupation by the British of the besieged city meant the end of the war, for he only enlisted till such time as Sir George White and his garrison should be released. He speaks of the spontaneous welcome given to them as "grand," though the sight of a friend who had been a big stout fellow when he had said "Good bye" in October, now hobbling up with a stick, his clothes simply hanging in folds, and his cheeks shrunken and hollow, nearly unnerved him. Another man whom my correspondent had known well, though quite young, looked twenty years older than four months before, and was just a wreck. The appearance of the men, and more especially of the women and children, proved how well founded had been the apprehensions of the relatives who refused to be comforted by the official statements "Food supply ample," and who had guessed all too well the straits to which the poor souls were reduced. He says, "For the last ten days before the blessed first of March came the rations had come down to half a pound of mealie-meal a man per day, which is a third of the ordinary amount allowed to a native. Had a serious attack been made upon the town there were only 2,000 men in a condition to fight, and they had been kept on full rations all the time in order that they might be in fighting trim to defend the camp. Even those who were not seriously ill were suffering from festered hands, the result of so little vegetable food. The privations had especially told upon the men from the 'Powerful.' As a rule sailors lead such an outdoor, breezy life that evidently they had found the confinement particularly trying, for they looked so wan, their noses and cheeks so thin, and their chins so sharply pointed. Most of them also were very 'down.' They seemed to feel so much the reduction in their numbers, thirty dead and forty very ill in hospital, many of whom might probably not recover."

The Carbineers were told to find quarters where they could, but as they had slept for six weeks without blankets or waterproofs or any extras, and no tents, they did not fancy they would be difficult to please. Some half-dozen men asked at the first house they came to if they might be allowed to lie down in the verandah. The lady offered them the dining-room floor if they preferred it when she knew they were relief men, but it was so warm that it was pleasanter out of doors. A little girl of eight came up to chat, a sweet, old-fashioned mite, but so white and frail-looking. Addressing one of the Carbineers she said, "Oh, sir, won't any more shells come into the town?" And the young fellow replied, "Never any more, dear," and with a touching sigh of relief the little one said "Thank God." Then, as if to show that notwithstanding all her sad experience she was still a child, she inquired wistfully, "Have you a weeny bit of the Queen's chocolate left? I haven't tasted sweets for months." You can imagine how glad that Carbineer was that he still possessed a fragment at the bottom of his pocket.

The war has, after all, cost us our Postmaster-General. The Duke of Norfolk was anxious to go to South Africa at the outset, but his application was refused, and it is rather curious that, having yielded to this decision, he should, at a much later stage of the campaign, have renewed it with success. As he also persisted in giving up his appointment at St. Martin's-le-Grand, Lord Salisbury had, of course, to find another chief of the department, and he has found him in the person of the Marquis of Londonderry. The new P.M.G. is not only a great landowner, and a polished courtier, who creditably filled the exalted post of Lord Lieutenant of Ireland; he is also a first-rate man of business, who has managed his coal mines with great advantage to himself, and presided over the deliberations of the London School Board with substantial benefit to the community. As he notoriously declined to accept a sinecure office under Government, one must conclude that he is really fond of hard work.

A compensation for the absence of excitement over this year's boat race was the presence of the sun, so often behind the clouds when the University crews contest their superiority upon the Thames. The hour, too, was a convenient one, so that the number of sightseers was a larger one than had been anticipated. I was told that the Cambridge crew was the best which was ever "set upon" the Thames, but I think that was an exaggeration. Anyhow, it was so superior to Oxford that at no time did the Dark Blues have "even a look in," as a schoolboy near me remarked, and when the end came we were not surprised to hear that it had been impossible to count the victory by lengths because they were so many. Oxford was nearly a minute later in passing the winning post than her opponent. They still, however, have a majority of eight wins against them.

### Important Points in Typhoid Nursing.

### EXAMINATION QUESTIONS FOR NURSES—RESULT OF MARCH COMPETITION.

The question was as follows: "What do you consider the most important points to be attended to in the nursing of typhoid fever?"

### The First Prize.

"Nurse Adèle" takes the first prize; her answer taken altogether is not better than several others, but she is the only competitor who points out the necessity of removing the patient from the source of infection.

### The Second Prize.

"Nurse Hancox" takes the second prize; her answer is very good, short, and to the point.

### Honourable Mention Awarded to Three.

Nurse Camilla, Nurse Mary, and Nurse Grace receive honourable mention. On the whole the papers are very good this month. There seems to exist a slight haziness of mind as to the amount of nourishment to be taken, and the form it should assume. It is hoped some light may be thrown on the subject in April.

### Nurse Adèle's Answer.

The first point in a case of typhoid fever to be considered is to remove the patient as far as is possible from the source of harm, which may be bad air, bad smells, bad feeding, and bad surroundings. The next essential point is to place him in a suitable position for treatment to have the desired effect. He must be kept strictly in a recumbent position in bed, and not allowed to turn or lift himself in the slightest degree without aid. As this enforced prone position is one that opens the way for bed-sores, together with the emaciated condition consequent on the fever and low feeding, the greatest care must be taken to prevent too long pressure on prominent parts by continually turning the patient from side to side; about every two or three hours may be sufficient. Suitable pads and water pillows should be used early in order to aid also in prevention of bed-sores. The next important point is that of feeding. The patient's strength must be maintained as much as possible by fluid diet and stimulants as ordered, to counteract the ravages of the fever. Nothing solid may be given on any account for at least three weeks or till the evening temperature is normal for a week, which time varies in every case. The usual dietary is four to six ounces of milk every two hours, diluted as required with lime, barley, or soda water. Strained tea may be given, and the diet gradually changed to jelly, light puddings, bread and milk, thin bread and butter, pounded fish and meat, as the patient gets better. Another point of the utmost importance is that infection is carried by the excretions, so that the stools must be always disinfected with very powerful disinfectants. Carbolic acid solution 1-20, perchloride of mercury solution 1-500, or perchloride of lime are good for the purpose. The stools must be always covered in being carried away, and if they have to be reserved for inspection they should be covered with lint or rag soaked in a strong disinfectant.

All linen connected with the patient must be disinfected before sending away. The sputum should be disinfected, and rags or lint used in lieu of handkerchiefs, which may be burnt.

A basin containing disinfectant solution should be always kept by the patients' bed for whoever is attending to him to dip their hands in. The hands should, however, be carefully washed as well as disinfected before eating.

Extreme watchfulness and observation are needed on the nurse's part so as to be able to report any symptoms of complications that may arise, such as pneumonia, peritonitis, hæmorrhage, perforation thrombosis, delirium, heart failure, &c. An accurate and regular report of the temperature, pulse, and respiration should be kept; it is usually taken every four hours.

Great attention is needed to keep the tongue and mouth clean; glycerine and borax or lemon juice and water are useful for this purpose.

### Nurse Hancox's Answer.

1. Feeding is of the utmost and first importance. The patient must be kept absolutely on light nourishing strained fluid diet. No solid matter of any kind is to be given till the temperature has been normal for at least seven days.

2. Rest and the recumbent position must be enforced, especially is this to be observed during the third and following weeks, as it is at this period typhoid ulcers have a tendency to slough, and the risk of perforation is greatest. Care must be taken to prevent hypostatic congestion.

3. Extreme care and patience with regard to cleanliness and the prevention of bedsores. As diarrhœa is a troublesome complication, this point will need unremitting attention, and a bedsore once formed will be most difficult to heal, and cause great pain and discomfort to the patient.

4. The next point is to prevent spread of the disease. All soiled linen must be immersed in disinfectant lotion. Bed-pans and utensils to be treated in same way. Stools must be covered with some strong solution of carbolic or hydrarg perchlor., and allowed to stand some little time before being emptied down the drain. Drains to be well flushed with plenty of water, and frequently with disinfecting fluid.

Points to be noted to the doctor: Temperature, pulse, and respiration; number and character of stools; if urine is passed; appearance and development of spots; amount of nourishment taken; hours of sleep. Nurse will also note the presence of curds either in stools or vomit, as these may cause fatal perforation.

Nurse to keep her hands perfectly clean, and finger nails short. She will also keep her hair well washed and brushed; take outdoor exercise, and good nourishing food.

### The Best Answer Sent In.

"Nurse Isabel" sends us much the best paper. She is disqualified for a prize through three previous successes. It is regrettable that her answer cannot be printed, for it would be valuable to all nurses, but 671 words exceeds the allotted number. I take this opportunity of warning all against too much "diffuseness." If a nurse is ever to be a teacher of others she must learn to condense valuable information—as it was expressed to the writer last week, "boil it down." The boiling pot is most useful.

### Question for April.

Describe how you would deal with a patient brought into hospital on a stretcher, and reported to have been shot in the side.

---

### Rules.

The competition is open to all. Answers must not exceed 500 words, and be written on one side of the paper only. The pseudonym, as well as the proper name and address, must be written on the *same* paper and *not on a separate sheet*. Papers may be sent in for fifteen days only from the day of the publication of the question. Failure to comply with these rules will disqualify the candidate for competition. Prizes will be awarded for the two best answers. Papers to be sent to "The Editor," with "Examination" written in the left-hand corner of the envelope.

N.B.—The decision of the examiners is final, and no correspondence on the subject can be entertained.

In addition to two prizes, honourable mention cards will be awarded to those who have sent in exceptionally good papers.

### The English Hospital, Haifa.

By a Special Correspondent.

At the foot of the headland of Mount Carmel, and overlooking the bay of Acre, lies its haven, the town of Haifa. Just above the town, on the slope of the hill, stands the English church, and, almost joining the church, the hospital. I do not think I shall ever forget the curious feeling which possessed me the first moment I saw it. Having come a long sea voyage, viâ Constantinople—in order to avoid the quarantine against ships touching Egypt—I was indeed glad to reach my journey's end. It was evening when the ship arrived at Haifa. The moon was shining brilliantly, and its soft light made the white Eastern houses look as though they were covered with snow. Two of "Cook's" men brought me and my luggage from the ship in a small boat to the landing stage. There, amid the usual din and noise of landing in the Orient, I was literally dragged ashore by friendly English hands. After my passport had been carefully examined by a disagreeable-looking Turkish officer, I was let through the custom house without—thanks to "Cook"—having one single article of my baggage inspected, and was at length "bundled" into a carriage with those members of the hospital who had kindly come to meet me. Having seen my trunks safely on the backs of two sturdy Arabs who kept close by the carriage, we proceeded without further delay along the narrow, dirty, uneven streets to the hospital; the so-called "drive" being nothing more than a ten minutes' jolt. As we reached the gates, I came face to face with a square, compact-looking house, upon the front of which—written in large wooden letters, painted black—were the words, *English Hospital.*

### First Impressions.

Everything seemed so still and peaceful, and mixed feelings of joy and sadness, hope, courage, and faint-heartedness, ran through my mind as I realized that this assuredly was my destination. Partly from a desire to use my training as a nurse for the benefit of whoever might come as patients to that odd-looking stone house; partly from a great wish to see the Holy Land, and—I must own—a natural love of adventure, had I left England and all that was very dear to me. My only hope was that the end might justify the means. It all seemed very weird and strange as I crossed the threshold that December evening, and I felt dazed and like a top that had been spun round and was not yet quite still. Needless to say, I was very glad to go to bed that night, and after a refreshing sleep, awoke the next morning to find I was beginning to realize my whereabouts.

After breakfast I was shown over the hospital. By day the building had a much less uncanny, but, happily, more alive and practical appearance than by moonlight. The house is built in a kind of half-Oriental, half-European style, which, to my mind, is not nearly so pretty nor so suitable to the country and climate as a native house. Instead of a white, flat roof, there is a bright red-tiled slanting one. The building has two stories. The ground floor forms the hospital proper, and the flat above, the nurses' residence. There are several verandahs, and all the rooms open into a marble hall.

### The Wards.

The men's two wards are on the right hand as one enters the hospital. These lead one into the other and hold, respectively, four beds. Next to them is the dispensary; a most up-to-date little room. Leading out of it is the doctor's room, and from this one passes into the theatre. The latter possesses all the necessaries, if not the luxuries, of modern antisepticism. It also has the advantage of a strong light from two large windows. Beyond the theatre are the women's two wards, each of them holding two or three beds, as occasion may arise. With the exception of the halls, all the floors are made of red tiles and the walls are whitewashed. The flat above where we live has one large hall, which we use as a dining-room. On either side of it are the doors of the nurses' rooms, and at one side is a large shady verandah.

Such is the extent of the temporary English Hospital at Haifa. That a larger, permanent one may soon be built is the hope of everyone connected with it. Of course, it is all very un-European, and at first strikes one as odd. But now, after a few months' residence here, I feel I should not like to have anything but white-washed walls, stone floors, and—instead of carpets—rush mats.

### The Staff.

Our staff consists of three English-trained nurses, one acting as housekeeper and head generally. We have a very clever German doctor, who gives his services, but who is, unfortunately, shortly returning to Germany. However, an English medical man has been appointed, and is, I believe, now on his way out to us. We also have the services of a good native doctor who, as well as attending to some of the in-patients, does all the prescribing at our town dispensary. We have a ward man—a kind of orderly—who, besides sweeping the men's wards, making the convalescents' beds, acting as interpreter, &c., is also our general factotum and bodyguard. A Jewess prepares the food for the Jews, who will not eat out of vessels defiled by Gentiles; food and utensils are kept sacred for their use. The same Jewess also sweeps the women's wards, and is responsible for many other duties, not the least of which is being washerwoman to the whole hospital.

### The Work.

When there is no night duty our actual nursing work is very light. But the days seem never too long, and we are not able to count upon long hours off duty. Some days we scarcely have any time to ourselves; another time we can have the day, or even two or more days, entirely free. The head nurse has often a weary time of it with the native servants. Notwithstanding all their good intentions, they are so utterly undisciplined that it seems well-nigh impossible to attempt to tran them. They have no idea of the value of time, no method, and no memories. Each meal has to be ordered separately, and just before it is necessary to begin preparing. The stores, too, are given out twice a day. We nurses do most of the cooking, the so-called cook being in reality nothing more than a kitchen-maid.

Housekeeping in this country is no easy task for an Englishwoman, especially when it comes to marketing, and consequently having to wrestle with this most difficult Turkish coinage. Napoleons are changed into majedis, majedis into bishlicks, bishlicks into piastres, piastres into metlics, paras, and sartoots. It is very perplexing. For instance, two half bishlicks are more than one whole bishlicks, and so on. One of our nurses is a qualified dispenser, and half her time is ocsupied in making up medicines. The third nurse has an indefinite position, and unless there is night work—which she always undertakes single-handed—or unless the head nurse happens to be away, in which case she fills the latter's place.

### Holidays.

In the very hottest weather the hospital is shut up for a month or six weeks, when the nurses have their annual holiday. But we are often able to have a few days at other odd times, which, in a climate like this, is really a necessity.

### The Nursing of Dysentery.

By a Nurse from the Tropics.

Every private nurse should certainly know something of the nature of tropical diseases. In the wide range of private practice "tropical troubles" are sure to crop up, either as distinct cases or as complications of other conditions. Undoubtedly, the nurse aspiring to war duty should understand how to care for a dysenteric patient. There will be large numbers of dysenterics returning from South Africa who will need skilful care. An exceptionally highly-trained nurse recently brought up a breakfast of fried bacon and mushrooms to her patient—a surgical case complicated with dysentery contracted in India—and she found her patient's horror very difficult to understand. The breakfast was perfectly orthodox from the standpoint of the surgical trouble. From the dysenteric point of view—which she did not stop to consider—the dish was fatally chosen. A good many nurses—untrained as British nurses often are in dietetics—make mistakes of this kind. It is not surprising that her patient lost faith in the infallibility of a nurse who could thus transgress, in spite of a long and admirable training, the very elements of tropical nursing. The trouble was that she had never learned anything on the subject. The existence of schools for tropical diseases shows that there is some necessity for training the medical profession in such matters. Therefore there must exist an equal reason that nurses should be instructed in the theory, at least, of nursing the diseases of other climates.

### No Two Cases Alike.

As a "case," dysentery is extremely interesting, for no two are alike. It needs good nursing, too, to prevent the patient from drifting into that hopeless and wretched condition known as chronic dysentery. The kind of nursing the patient gets goes far towards deciding the question. The after effects are more to be dreaded than the disease itself, hence much depends on skill and care. Tropical dysentery is a very different disease from the so-called dysentery of temperate climates. To develop dysenteric symptoms in a healthy climate shows that the system is in a very bad condition, and such cases are apt to linger on in a weary state of chronicity. In the tropics the disease takes a more acute and malignant type. It is a water-borne disease, and though not communicable by contagion, it often appears in epidemic form. It occurs both in low lying malarial districts and in hill-stations.

### The Origin.

Very little is known of the origin of dysentery. Polluted water, bad drainage, dust and sand swallowing as among the South African troops, chills, bad food or not enough of it, as in Ladysmith, are all exciting causes. An irritating diet, a constant use of hard biscuit, as in Army and Navy, and tinned foods, and all unhealthy conditions in general, tend to produce dysentery.

In persons with a tendency to inflammations of the mucous membranes a slight abdominal chill will cause a dysenteric attack. Going from the plains to tropical hill stations is a common cause of the disease, especially as the "healthy hill-stations" of India and Ceylon are often in a shocking state from the sanitary point of view.

Infants and children are particularly liable to it, and the onset of the disease is marked in all patients by chill, abdominal pain, and tenderness with straining and constant desire to pass a motion.

### The Symptoms.

The disease may come on suddenly or slowly. In the slow, gradual form it is often taken for diarrhœa; the patient does not examine the stools, and keeps up and about.

The colon is the seat of the trouble, the mucous membrane of this being merely inflamed and highly congested or going on to a badly ulcerated state. The condition is not dissimilar to that of typhoid, but the dysenteric ulcers are much larger and more virulent, and are chiefly situated in the sigmoid flexure and descending colon, and rarely in the small intestine as in typhoid. Some 12 to 16 inches of the colon may be thickly studded with ulcers varying in size and depth. A dysenteric ulcer patch may cover several inches of the intestine, and the ulcers tend to burrow deep into the mucous membrane.

When the ulceration is near to the cæcum there is more abdominal pain and griping; if close to the rectum more tenesmus is present.

The characteristic motions of dysentery consist chiefly of mucus, tinged, streaked, or spotted with blood, scanty and passed with extreme pain and tenesmus. Probably before this stage has been reached several diarrhœal motions, with or without blood, have been passed. In the later stages little fæces are present, the entire motion being mucoid mixed with blood. From eight to 12, or more, may be passed in the 24 hours. Usually there is very little fever. Even in the most acute case the temperature goes up only one or two degrees. Chill and nervous depression are marked features.

A very lowered vitality and reduced power of resistance accompanies the dysenteric state.

### Nursing.

The first nursing measure is to put the patient to bed, remove as many pillows as he will allow, so that the position may be really recumbent, and to keep him and his room warm and free from draughts.

If the patient be allowed to sit up, this position throws an added congestive strain on the bowel which is already congested and inflamed. It will be a constant struggle of nurse and patient to induce the latter to pass the motions lying down. He will long to be allowed to sit up for this purpose, believing that a sitting posture relieves the intolerable straining and tenesmus. As a matter of fact, the passage of the motions while lying down is in itself a relief of tenesmus and congestion. But it is very difficult at first to persuade the patient of this. Great rectal cleanliness must be observed throughout the case. After each motion, the anus should be gently wiped with soft absorbent cotton, linen, or, better still, pieces of old many times washed flannel. These should be at once burnt. The rectum should then be bathed with *hot* water and a small quantity of eucalyptus ointment gently applied to fundament and surrounding parts. Unless these precautions be adopted, the skin will become painfully sore and excoriated and tenesmus be largely increased by the general irritability of the parts. If the perspiration between the buttocks be excessive—as is sometimes the case—an aseptic powder should be freely used, otherwise an irritating eruption is liable to follow.

Pledgets of cotton-wool wrung out of very hot water and applied over the anus, afford great relief to the tenesmus. A loose abdominal binder of flannel or silk is an essential in dysentery. Medical opinions are divided as to whether the flannel cummerbund is advisable for constant wear in the tropics. Some doctors call the abdominal belt a "dysentery trap." Others advise the thickest possible woven belt to be worn at all times and seasons in the tropics. In a damp, hot climate, the belt undoubtedly acts as a poultice, and often produces such an eczematous, itching skin eruption—or poultice rash—that its use has to be discontinued.

I have found that a silk cummerbund next the skin is infinitely better both for dysenteric patients and for those who wish to prevent themselves from becoming so. Even with the silk abdominal binder, the nurse will need to powder the skin under it once or twice a day with starch powder or Fuller's earth.

Some doctors, however, prefer their dysenteric patients to develop an abdominal rash, as experience seems to show that such cases do best.

Abdominal pain and tenderness is relieved by hot flannel or spongio-piline fomentations. Turpentine stupes are preferable when the skin of the abdomen is not sore and eruptive.

Two Classes of the Disease.

Dysentery is divided into two classes, the catarrhal and the ulcerative. In the catarrhal type, although the mucous membrane may be intensely congested and bright red with inflammation, no ulcers are formed. The catarrhal kind of dysentery is apt to be mild and to resolve itself into an early convalescence. Ulcerative dysentery, on the other hand, is a much more serious disease: offensive sloughs—showing that the ulceration is deep—are often thrown off in the motions and the condition of the patient may become critical. Neglected ulcerative dysentery is apt to drift into the serious and distressing condition of chronic dysentery.

Gangrenous dysentery is a further and nearly always fatal development of ulcerative dysentery. The patient is collapsed and delirious and looks very much like a cholera case. Every variety of dysentery is apt to become serious in patients accustomed to much alcohol. For this reason the disease is much more fatal to men than women, and is more feared by masculines in the tropics than by feminines. The nurse must in all cases watch for hæmorrhage, which may be of a serious nature in ulcerative dysentery if the slough sloughs down to a fair-sized artery. Perforation is rare, though possible.

### The Consumption Sanatoria, Bridge of Weir.

By a Nurse.

So much has been written lately about the open-air treatment of the diseases of the lungs that a brief account of the past year's work in the sanatoria at the Bridge of Weir may be interesting. Bridge of Weir is about ten miles from Glasgow, and this Sanatorium occupies an ideal spot, nestling among the Renfrewshire hills, far from the noise and smoke of that great city. It contains accommodation for twenty-six patients, who are in charge of a matron and three nurses. The patients, except a few new-comers, spend all day out of doors, walking, sitting, or reclining on lounges, apparently happy and content. They have also little pavilions scattered throughout the grounds, which are utilised when the weather is wet and stormy. According to the last report 22 per cent. of the cases admitted had already been cured, and 44 per cent. had been so greatly improved that they might be classified under what the Germans call relative cures. Two of the patients I spoke to had put on weight to the extent of three and three and a half stone respectively in six months. The Sanatorium is purely philanthropic, and expenditure for food alone exceeds £2 per week for each patient. Most of them belong to the artisan class. Mr. Quarrier, the founder and director of the sanatorium, strongly disapproves of sending out collectors or asking in any way for money to maintain it. He simply makes known his wants, and they are always supplied, though ofttimes from day to day. The matron and nurses are very enthusiastic, and delighted to show visitors over; so if any of the readers of the "Nursing Mirror" are in that locality I can guarantee them not only an interesting but a most enjoyable afternoon.

### Nursing the Wounded at Naauwpoort.

By an Army Nursing Reserve Sister.

How I wish I had time to write all there is to tell you. We have been so busy this week that we have taken very little off-duty time, and when we did we were too tired to write. On Sunday last over two hundred patients arrived, so the four of us had a busy time. The majority of these had been wounded at Paardeberg, and what they went through makes one's heart ache to think of. Many of them had not seen a bed for four months, and some not even a tent for two. Their gratitude is quite pathetic. Most of them have lost their kit, and those who have not have little more than rags to wear.

We have not nearly enough things to give the men. I have been wiring to ——— to-day for socks and shirts. The hospitals near Cape Town are well supplied with everything, but here, being so far up country and new, we have not half what we want. We have over 500 patients, and others constantly arriving. "Tam o' Shanters" they love; shirts and handkerchiefs are very acceptable, and cigarettes. We gave away a few of ———'s handkerchiefs the other day, and the men's faces were a study. They were so pleased, and everybody of course wants one; some are having them framed, others are sending them straight home to their wives and mothers to be prized and taken care of. I spent 5s. in matches for my patients the other day. They have had to give 6d. for a penny box up at the Modder. Here I paid 5s. for a gross.

We have soldiers of all regiments, and many of them Highlanders. On Wednesday a number of seriously wounded came in. It is extraordinary how brave and good they are, and how quickly their wounds heal. There were eighty stretcher cases among these, and we were much hindered in moving them from marquee to marquee by a terrific thunderstorm. When the storm abated a little we all worked as hard as we could to settle everyone comfortably for the night. Sister D—— and I have had a very heavy day, but the life is entirely different to that in a civil hospital, and I am most glad of the experience. We are on our feet all day, but living as we do almost out of doors is far healthier than being shut up in a stuffy hospital. We are on duty from eight a.m. to eight p.m., with one hour off from one to two o'clock, and again from three to six. These are nominally our hours, but of course we have been too busy lately to keep to them. The P.M.O. has sent for ten more sisters, so when they arrive the work will be lighter. I am very glad I came out, as I am very happy in my work, but I often wish I could do more. I am making a small collection of bullets and bits of shell, &c., to bring home from my grateful patients. We were presented with some chickens taken from the Boers a few days ago. One of them laid an egg this morning—a great excitement, as they cost sixpence each here.

We are getting many cases of enteric sent to us, I am sorry to say. This is not to be wondered at considering the water the poor men have had to drink, and often they have been 30 hours without getting a drop. I am ambitious enough to hope we may be so lucky as to get into Pretoria when it is relieved.

### To Nurses.

We invite contributions from any of our readers, and shall be glad to pay for "Notes on News from the Nursing World," or for articles describing nursing experiences, or dealing with any nursing question from an original point of view. The minimum payment for contributions is 5s., but we welcome interesting contributions of a column, or a page, in length. It may be added that notices of entertainments, presentations, and deaths are not paid for, but, of course, we are always glad to receive them. All rejected manuscripts are returned in due course, and all payments for manuscripts used are made as early as possible at the beginning of each quarter.

### The Army Nursing Service Bill in the United States.

BY AN ENGLISH NURSE IN AMERICA.

THE principal members of the nursing world in the United States are at the present time directing their energies and using all their influence to secure the passing of the Army Nursing Service Bill, a Bill to provide for the appointment of graduate female nurses in Military and Post Hospitals in this country.

THE BILL.

The following are the provisions of the Bill:—

SEC. 1.—Be it enacted by the Senate and House of Representatives of the United States of America in Congress assembled,

That from and after the passage of this Act, women nurses in the proportion of not more than 10 per cent. of the number of sick and wounded in General and Post Hospitals of fifty (50) beds and upwards, shall be employed by, and constitute the Women's Nursing Service of, the Medical Department of the Army.

SEC. 2.—That there shall be a Superintendent of Women Nurses in the Army, who shall be a woman graduated from a general hospital training school for nurses, having a course of instruction lasting not less than two years, and who shall be appointed by the Secretary of War.

That the salary of the Superintendent of Nurses shall be two thousand ($2,000) dollars per annum.

SEC. 3.—That the Nurses in the service shall be graduates of general hospital training schools having courses of instruction lasting not less than two years; that they shall be appointed by the Surgeon-General of the Army under such regulations as may be approved by the Secretary of War; that they shall receive forty dollars per month when on duty within the limits of the United States, and fifty dollars per month when on duty outside of the limits of the United States. That a Chief Nurse may be appointed for every hospital where there are five or more nurses on duty; that the salary of a Chief Nurse shall be seventy-five dollars per month within the limits of the United States, and eighty-five dollars per month outside of the United States.

That, in addition to the salaries above-mentioned, the Superintendent of Nurses, and each Nurse in the Women's Nursing Service, shall be entitled to transportation and necessary expenses when travelling under proper orders; and provided, further, that Nurses and Chief Nurses shall be entitled to quarters, subsistence, laundry for uniforms, medical attendance, nursing and medicines during illness, and that they may be granted such leaves of absence without loss of pay as the Secretary of War may authorize.

SEC. 4.—That the number of Nurses necessary for the immediate establishment of the Women's Nursing Service may, upon the passage of this Act, be appointed by the Surgeon-General.

SEC. 5.—Provided that nothing in this Bill shall be construed to prevent or to limit the power of the Secretary of War in time of war or of national disaster to avail himself of duly-qualified Red Cross Nurses (termed "sisters") or of nurses of other worthy societies or associations.

WHY THE BILL WAS INTRODUCED.

The recognition of the need for the existence of a corps of nurses specially trained in army nursing, and with a thorough knowledge of the routine work in military hospitals, was forced upon the public mind during the recent Spanish-American war, when, in consequence of no provision having been made for an adequate supply of nurses, the deaths from disease and lack of care far exceeded those from the effect of bullets. At first it was decreed that no female nurses should be allowed at the seat of war or in the military hospitals. "There were twenty well-trained orderlies, who would be amply sufficient." But soon the cry came to Washington from the already overcrowded camp and other hospitals for nurses, more nurses. Volunteers, who, however, had to be either native or naturalised American citizens, were asked for, and nobly was the appeal responded to by nurses from every part of the Union. Many gave up lucrative and responsible positions to care for sick and wounded fellow-countrymen. But still more were needed; the stern answer that none but Americans need apply was rescinded, and soon the twenty well-trained orderlies were supplemented by seven hundred female nurses. But much of their labour and skill was wasted, because there was no recognised head to guide and direct their work in the different hospitals. There were graduates with various dispositions and qualifications from all kinds of training schools, and having been trained in different methods, each to a certain extent planned her own work and controlled her own actions. Those who showed executive ability became by natural selection the supervisors of the others. Many of the accepted volunteers had had no previous training, but were put on the nursing staff at the instance of some friend or relative who possessed, as it is aptly called, some "political pull." These enthusiastic and well-intentioned amateurs were not the best nurses for men very ill with dysentery, typhoid, malaria, or wounds, and needing well-trained, experienced care as regarded their nursing, treatment, diet, and administration of medicines.

THE PROMOTERS OF THE BILL.

Mrs. Hawley, who as Miss Horner came in 1884 to Philadelphia with Miss Alice Fisher, from England, to take charge of the nursing in the City Hospital, Philadelphia, is one of the chief promoters of this Bill. Owing to her untiring efforts and the hearty co-operation of the superintendents of the largest training schools of this country, a committee has been formed to further its passing.

By meetings, resolutions, delegations, and personal interviews, they have represented to the Secretary of War, the Surgeon-General, the Senators, Representatives, medical profession, and the public that it is neither just nor economical to compel sick or wounded soldiers to undergo needless hardships, suffering, or risk of life. Yet such evil results must frequently occur unless military hospitals of fifty beds and upwards are made equal in all essentials to civil hospitals of the best grade. One of these essentials, as the Spanish-American War has emphasised, is the providing of an organised body of trained women nurses. This provision for the Army should not again be deferred until the emergency actually occurs; nor should it be left to private patriotism and charity; nor should it depend on the views and actions of any individual official. It should have the sanction and permanence of law.

To this end the foregoing Bill has been prepared for the establishment of a nursing service in the United States Army, which has been introduced at the present session of Congress, the chief features of which are the development under efficient supervision of a service consisting of a superintendent, who shall be a graduate of a training school for nurses, and of conspicuous executive ability, and a corps of carefully-chosen nurses—such a service as is in existence at Netley and other English military hospitals.

It is now desired to arouse national interest in the passage of this Bill, and to influence members of Congress to give it their support. Civil hospitals have demonstrated beyond question that only by putting the wards in charge of trained women nurses can the best results be achieved, and unless military hospitals do the same we shall be open to the reproach that we do less for our soldiers than every State does for its alien paupers.

### Where to Go.

CRYSTAL PALACE.—Great sacred concert, Good Friday, at half-past three. Madame Ella Russell, Miss McIntyre, Miss Clara Butt, Mr. Edward Lloyd, and Mr. Santley will sing.

### The Nurses of the Imperial Yeomanry Hospital en route to the Cape.

By One of Them.

It is just three weeks since, despite the cold and wet, a cheery, cheering crowd collected at Southampton to wave a farewell to the "Guelph" and her passengers. The soldiers swarmed fore and aft and gave back cheer for cheer, while the rest of us watched the scene and wondered what would have happened before we again returned to the dear homeland. A few days later a limp, dejected lot of people gradually assembled on the decks. One wondered what had become of the life and enthusiasm lately displayed by them. But the Bay had been very unkind, and, not content with giving us a bad time, had also retarded our progress, so that we arrived at Teneriffe on the 24th, a day late. There, again, we lost time, as the coaling took at least six hours longer than was anticipated, continuing well into the night—the deafening shouts and yells of the men accompanying the performance. We were pleased to get ashore again, though we found nothing particularly exciting about the place, people, or the things they had for sale. As we approached the island it presented the appearance of a number of irregular cone-shaped peaks one behind the other, any spot of cultivation being cut in terraces on the sides of the hills. There appeared to be no trees whatever, and a drive taken later did not dispel the first impression formed as to the barrenness of the land.

### At St. Helena.

On March 7th we spent a delightful day at St. Helena. This island appeared even more rocky and barren than Teneriffe before landing, and looked a quaint spot with its handful of houses situated in a deep, narrow valley between the hills. But we found trees and groves of bananas, whose cool shade and green leaves were distinctly refreshing. Our first thought was to post our letters, and we wended our way to the primitive little post-office, where the pretty little brown-faced, brown-eyed post-mistress was looking distracted, though interested and amiable. Stamp collectors could not resist this opportunity of adding to their store when so many surcharged stamps lay temptingly before them. The next move was to arrange a start for Napoleon's tomb and the house in which he spent his last days; but we were disappointed, as the few ponies and fewer vehicles of which the island was possessed had been snapped up by those who had been so fortunate as to get ashore first. Several brave spirits attacked the hills, despite the scorching sun, and after a hot and weary walk of an hour and three-quarters arrived at the tomb and saw the roof of the house in the distance.

### The Military and Civil Hospitals.

The rest of us meanwhile sauntered about and visited the military and civil hospitals, in both of which our party received a warm welcome. In the latter there were three nurses, who each had come originally from South Africa, one having left Johannesburg five months ago with the first rush of people from that place. The hospital was cool, the wards pretty—altogether it was a tempting place in which to rest, and once more indulge in a cup of tea made on land. One of the patients was a picturesque old black woman, who had been at one time a slave, and still bears on her back the marks of many beatings. Some of us had gone ashore with romantic visions of fruit lunches, but, alas! there was little or no fruit to be had, and one of the sisters, returning in triumph with a basket of oranges, was rather chagrined to find that they had been unloaded from the "Guelph" a few hours previously. And now we are looking forward to arriving at Cape Town on Wednesday next, the 14th. We are eager for news, and longing to be at work. We were delighted while at St. Helena to hear the good news of the relief of Ladysmith, the surrender of Cronje and his men, and the restoration of communication with Kimberley.

### Drilling the Sisters.

We have on board 800 men and 24 officers of various volunteer regiments, and our chief entertainment has been in watching them at their drill and exercises, their boxing and sports, cricket and shooting. The officers ranged at intervals along one side of the hurricane deck shoot with revolvers at corked bottles, which, thrown from one end of the vessel, floats past a target for each one in turn, while the Tommies do their shooting from the stem of the vessel, their target being a box which is arranged to float at a certain distance. The sisters are drilled by the Gordon sergeant for half an hour before breakfast each morning, which they very much appreciate, regular exercise being so essential on board ship. The first morning the sergeant's stern command of "silence," and later his shout of "squad" as the preface to an order, created much amusement.

### How the Tommies Amused Themselves.

In the beginning the Tommies had many informal concerts among themselves on their lower deck. They generally commenced with pathetic songs of farewell to sweethearts, homes, and the old folks there, brisked up and got enthusiastic over a song like "Tommy Atkins," and then the announcement of "Avenge Majuba" brought down the house. Whenever there was an approach to a refrain or chorus they all joined in, and with such a body of voices it sounded well. Latterly the concerts have assumed a more formal character, with programme and audience. Tommy scrubbing his khaki with all sorts and conditions of brushes from a broom to a nail brush, Tommy getting his hair cut, and Tommy showing how he could dance—in which last the Gordons show great dexterity and lightness of foot, and won much applause—all contributed an endless variety of things to watch. Many of the men have been inoculated against typhoid since coming on board. It was touching to see their comrades carrying them on deck, gently turning them, giving them drinks, &c. We hope to just catch a mail at Cape Town through our commanding officer franking our letters, thus preventing the delay of waiting for stamps.

### Royal Orthopædic Hospital.

The following entertainments have recently been given in this hospital to the nurses and patients: On March 2nd Miss E. Josephine Troup kindly sang and played, concluding her entertainment with the well-known recitation, in character, "Mrs. Brown at the Play." The varied experiences of that lady on visiting a theatre for the first time were humorously presented by Miss Troup, who kept her audience in laughter throughout. On March 30th the following ladies of the Royal Academy of Music kindly gave a concert: The Misses Ethel P. Cave, Annie and Sallie Bartle, Lizzie Davies, and Daisy N. Hansell. This was the third occasion on which, through the kindness of Mr. F. W. Renaut, the secretary, the students had visited the hospital, and the talent displayed by those ladies was highly appreciated. The programme consisted of violin solos by Miss Hansell, solos and duets by Miss Bartle (soprano) and Miss Davies (contralto), Miss Cave accompanying and also rendering several pianoforte pieces.

### Novelties for Nurses.

### A PERFECT SHOE.

There are few nurses who do not some way or other suffer from their feet. The constant standing, the polished boards or stone staircases, all in time tell a tale more or less deplorable. Flat feet, tender feet, swollen feet, do we not know them only too well. This condition of things can be greatly ameliorated, however, if nurses would pay a little more attention to the shape and quality of the shoes they purchase. Cheap shoes are always the dearest in the end. They lose their shape after one or two days' wear, and are of no support to the foot. The heels, too (which look so smart in the shop window), very quickly tread over, causing unnecessary tension on ankle and instep. A shoe destined to cope with all these evils has just been brought out by Messrs. Gooch's Stores (67, Brompton Road), from the design of a trained nurse, and bears the name of "The Princess Christian Ward Shoe." Built on sound scientific principles, this delightful shoe is made in the softest glacé kid and shaped exactly like a smart walking shoe, neatly laced up the front. The lacing, however, is purely ornamental, as on either side of the lace, the shoe is provided with the most ingenious little gussets of stout elastic, which permit of the shoe being slipped on or off, without any of the delay and trouble inseparable from the actual lacing or buttoning. Think what a saving of time this must be to busy professional women of all classes, and last, but not least, to many women the disagreeable necessity for stooping is entirely done away with. In addition to this speciality, the shoe is provided with all the other advantages possessed by Messrs. Gooch's Samaritan Ward Shoe. The instep arch supporter, and the square sensible military heel, rendered noiseless by the insertion of a small wedge of indiarubber, all combine to make "The Princess Christian Ward Shoe" as perfect as possible. This shoe, which is equally suitable for out-door wear, is procurable for the modest sum of 12s. 9d. and can be made in specially prepared black or tan leather for colonial wear.

### SPRING NOVELTIES.

### At Messrs. Garrould's.

A visit to the establishment of Messrs. Garrould, in the Edgware Road, is always an interest and a pleasure. Every variety of requirement, from the cradle to the grave almost, seem to meet with attention, and fastidious indeed must that purchaser be who cannot find everything she wants in one or other of the numerous departments of this celebrated house. Nurses will find a never-ending assortment of uniforms, plain and coloured linens (and how beautiful some of the new shades are), caps of the most bewitching design, lingerie of the whitest and finest, and the most gracefully cut cloaks imaginable. We are glad to see that there are signs of increasing refinement in all these matters; there is distinctly a little less tendency towards flounces and furbelows, linings of some pronounced and startling hue, and exaggerated strings and collars, than was the case a year or two ago, and we rejoice at it. There is no doubt that as nurses realise that the charm of uniform consists in the absolute neatness and freedom from exaggerations of any sort, the demand for the other class of articles will go out. Absolute simplicity, combined with excellence of cut, is what the refined nurse should aim at. Jingling chatelaines are also, we are glad to observe, going out of fashion, and are becoming gradually superseded by the equally useful but more suitable wallet. Of these Messrs. Garrould has a most fascinating assortment, many of them being dustproof and consequently aseptic. Dear little silver watches, with the Geneva cross in red enamel on the back, will be hard to resist, as they are very inexpensive, and guaranteed to go well. In some cases they are provided with a second hand, which is always a useful addition. The "Tikord" linen for aprons maintains its excellence as one of the most serviceable articles ever manufactured, and we should advise our readers to give it the preference over all other makes. There are also some charming designs in zephyrs and Halifax, the latter being a material of stouter make than the former, and consequently better adapted for rough wear. The tea room is still a great attraction, and has proved a boon to numberless weary nurses.

### At Messrs. Egerton Burnett's (Limited).

Messrs. Egerton Burnett (Limited) are to be congratulated on their large and varied assortment of spring novelties. Each season some original and striking design is to be found in the liberal assortment of patterns with which they delight the eyes of their numerous customers. So varied is the choice that every variety of taste is consulted in the designs submitted. This season there are some lovely new shades in blue and rose colour, nor is now the favourite khaki absent from the selection. There is a fascinating shade somewhat resembling putty which would prove admirable for cycling purposes, as it would not show the dust and always look cool and smart. It is manufactured in Amazon cloth, and also in a pretty twilled cotton material. The silk-faced lawns and zephyrs are simply charming, and make one long for the warm weather as an excuse for an investment. It would take too long, however, in the limits of an article like the present to do more than merely glance at a few of the most striking of the fabrics before us, and we must content ourselves with a description of those materials more immediately interesting to nurses. We can most strongly recommend the finer twilled serges which are offered in both navy and black. They strike us as being eminently serviceable, and are guaranteed not to shrink. They are suitable alike for cloaks and dresses. There are also some pretty shades of grey in both serge and other varieties of woollen material, as well as the ever-favourite Zephyrs and Halifax. There is a lovely silver-grey, silk-faced Zephyr, which would look well made up, and only costs 1s. 4d. a yard. The apron linen is also a good line, and is excellent value for the money. We desire to call special attention to the tailoring and dress-making department, which has proved so successful in carrying out the requirements of Messrs. Egerton Burnett's numerous *clientèle*, and which is a great saving both of time and trouble. By a simple system of self-measurement the most surprising results are assured, and the cost in each case is most moderate in comparison with sending the material to be made on arrival, to say nothing of the trouble of going to be fitted. The New Puttee, "Fox's Patent," is to be had for 5s. 6d. without the spat, and is much to be preferred in cold weather to the ordinary gaiter. It is procurable in black, navy, and all mixed shades. Bundles of charity serges can be had on application, and there is a large selection of blankets, coloured rugs, and Austrian blankets, suitable for charitable institutions. Our readers will be well advised to send for the price list and box of patterns, which are forwarded post free to any address. We do not think that they will be disappointed.

### A USEFUL INVENTION.

Dr. Alexander Duke is to be congratulated on a new form of vaginal douche tube which he has just brought out, and which certainly is a great improvement on those in ordinary use. It appears to be very searching and thorough in its action, the tube being surrounded by unrustable flexible wires, which can be bent with the fingers to the shape of the vagina, the walls of which they keep apart while the tube runs the whole length, flushing out the organ from above downwards. The perforations at the top of the tube through which the fluid is forced, are so placed that all contact with the vaginal walls is rendered impossible, the advantages of which must be obvious. In this respect it differs from any other form of vaginal douche. The appliance, as shown in the accompanying illustration, is provided with an improved form of receptacle for waste, which not only is more comfortable for the patient, but effectually prevents any wetting of the person or bed. An exit is provided for the waste fluid through a rubber tube attached, the end of which can be directed into a slop-pail or other convenient vessel. This ingenious invention can be inspected at the makers, Messrs. Coxeter and Sons, Grafton Street, London.

### A NEW CUFF FASTENER.

Dr. Gidley draws our attention to a new fastener for cuffs worn outside the sleeve. The elastic band which encircles the sleeve under the cuff can be procured made of nickeled steel or oxylonite from G. Brooks, ironmonger, Cullompton.

### A CHOLERA-BELTED COMBINATION.

At Messrs. Penberthy, the well-known establishment for underclothing, hosiery, gloves, &c., a most useful garment can now be obtained. It consists of a beautifully shaped and finished combination into which a cholera belt is woven. The advantage of such a garment is apparent at once. The belt must remain in place, clumsiness is avoided, and trouble dispensed with. Four sizes and three grades of warmth are supplied. The quality of the elastic woollen materials employed is splendid. The finest variety is soft and fine as silk, and especially suitable for warm climates. Nurses and other ladies who are going abroad should certainly supply themselves with these excellent garments from Messrs. Penberthy, 390, Regent Street, and they will be found equally invaluable to those who have been affected by dysentery and other tropical complaints, or require extra warmth in the region of the abdomen.

### The Nurses' Bookshelf.

[We invite Correspondence, Criticism, Enquiries, and Notes on Book likely to interest Women and Nurses. Address, Editor, The Hospital (Nurses' Book World), 28 & 29, Southampton Street, Strand, London W.C.]

What to Do in Emergencies. By Dr. Andrew Wilson. Edited by "Isobel," of *Home Notes.* (London: Pearson. 1900. Sm. 8vo., pp. 180. 1s.)

Dr. Andrew Wilson has a deserved reputation for the rare faculty of being able to express simply difficult and technical matter to the unlearned. In this volume he sustains this reputation completely. It would be found a good, cheap text-book for ambulance classes and similar methods of instruction, and could be usefully put into the hands of any intelligent young man or girl. The space given to fractures and dislocations is not unduly large, as it often is in such manuals, and the teaching given is less pretentious than usual. It is unsafe, however, to mislead a person untrained in anatomy into the delusion that it is easy to distinguish a sprained joint from a dislocation, and it is scarcely sound advice to suggest that any untrained person should apply pressure bandages to a sprained joint. There is a full chapter on poisoning, which should however have included remarks on antipyrin and sulphonal poisoning. The remarks on chronic lead poisoning might with advantage be omitted. The style of writing is a little staccato. Such sentences as "The treatment of sprains is an important matter, this for the reason that sprain is a frequent accident," could easily be better arranged, and such sentences are freely sprinkled through the book. We can recommend this manual without hesitation.

Amateur Gardening for Town and Country. Vol. XV. (Published at the offices of "Amateur Gardening," 148 and 149, Aldersgate Street, London, E.C. 1899.)

Many periodicals are now devoted to various aspects of gardening, and it is not a little astonishing how they all manage to find new and interesting matter for their readers. Prominent amongst these magazines is "Amateur Gardening," of which we have received the fifteenth volume. We can safely say that it would be difficult for more information to be contained within the limits of its pages than we have discovered there—information on the most widely diverse matters affecting a garden which cannot fail to be of interest as well as of practical use to those who love to grow and tend their own flowers. Besides a large number of articles dealing with roses, chrysanthemums, and other plants, as well as others treating of soils and other topics, every week contains several columns of most valuable matter in the shape of answers to correspondents who are in difficulty over some problem or other. When to the excellent character of the information given we add that the work is illustrated, not only by plain figures, but by very good coloured plates, it will be seen that "Amateur Gardening" is a very cheap and good magazine, and one which should interest all gardeners, whether their plots be large or small.

### Minor Appointments.

Chalmer's Hospital, Edinburgh. — Miss Margaret Hamilton has been appointed Charge Nurse. She was trained in the Dumfries and Galloway Royal Infirmary, was charge night nurse of the same institution, and has since been charge nurse at Kirkcaldy Hospital.

Victoria Hospital, Kingston-on-Thames.—Miss Jessie Caldwell has been appointed Charge Nurse. She was trained at St. Thomas's Hospital, and has since been on the nursing staff.

Abergavenny Asylum.—Miss M. L. Sabin has been appointed Head Nurse. She was trained at the Oxford County Asylum, and for some years since has been at the Dorset County Asylum.

### Everybody's Opinion.

[Correspondence on all subjects is invited, but we cannot in any way be responsible for the opinions expressed by our correspondents. No communication can be entertained if the name and address of the correspondent is not given, as a guarantee of good faith but not necessarily for publication, or unless one side of the paper only is written on.]

### SOME OF NURSES' DIFFICULTIES.

"Two Nurses of an East County Hospital" write:—We think that it may interest some of your readers to know one of the various difficulties which nurses have to contend with in their work. At present we have a patient who made an attempt on his life by cutting his throat, so badly that he has entirely lost his vocal powers. In order to make us understand what he wants, he writes it down. To read it is a hard task, as the following will show. We have copied these requests off the scraps of paper he is continually pushing into our hands—(*a*) "thear is monthing on myne chist." (*b*) "new peace or leant, has myne his geting durtir." (*c*) "when i caugh mi throt is liked fire, hot like them art burne." (*d*) "it his heard wark fer me to get mi brath. (*e*) "mi wyfe is in the feaver ospittle, and wone of the litle wones, and wone at mi motthers with the feavir." (*f*) "i knowed that men, which lied hover thear." In case the above sentences should need any translation, we send the following key—(*a*) There is something on my chest. (*b*) New piece of lint, as mine is getting dirty. (*c*) When I cough, my throat is like fire, hot like the heartburn. (*d*) It is hard work for me to get my breath. (*e*) My wife is in the fever hospital, and one of the little ones, and one at my mother's with the fever. (*f*) I know that man over there.

### A WARNING.

Mr. John Odling, secretary of The National Waifs' Association (Dr. Barnardo's Homes), says:—Apropos of the letter in your last issue under this heading, may I mention that a lady visitor answering the description given by your correspondent as to being gentle and refined, called recently at Her Majesty's Hospital, in Stepney Causeway, and at the Infants' Creche, representing herself as the wife of a medical man in Reading, and much interested in the establishment of a Creche in that town. After inspecting the institution, and making several enquiries about our methods, she made the supposed discovery that she had lost her purse and was without means of defraying the cost of her return journey. She succeeded in borrowing of a number of the staff the sum of 10s., promising that the money would be refunded by return of post. Since that time nothing has been heard of her, but enquiries have elicited the fact that the professional gentleman named by her as her husband is unmarried. It would be well that your readers should be on their guard against the unscrupulous methods adopted by this really fascinating visitor.

### PROMOTION IN INFIRMARIES.

"Nurses' Friend" writes:—In a recent copy of the *Marylebone Times* I read in connection with the appointment of an assistant matron to the St. Marylebone Infirmary, that the Infirmary Committee recommended, subject to the Board's approval, that advertising for candidates be dispensed with, and that Miss Henrietta Henderson, aged 28, be appointed first assistant matron. Mr. Harrison—one of the said committee, I presume—"did not think if they advertised they would get a better candidate, and without depreciating the services of any of their officials, there was not one they felt justified in proposing for this particular post, nor had any of them applied for it." From the above it is evident that an outsider has been appointed over the heads of the existing staff, and for this step two reasons are given. First, there was not one of the staff they felt justified in proposing for the post. Does this mean that out of the large staff necessarily employed in such an institution, not one of them was competent to undertake the duties of assistant matron? Surely not. But the gentleman himself is evidently dissatisfied with this statement, for he makes haste to add that none of them applied. But why didn't they apply? How could they do so if they were n't informed in some way that applications would be considered? But I need not go on. It was clearly a foregone conclusion that a stranger—and doubtless to a number of the staff a junior—should be placed over them. Now, sir, I have no animus towards the lady chosen—on the contrary, only good wishes for her in her new sphere, but, at the same time, as a friend of nurses in general, and of infirmary nurses in particular, I think it a pity when a Board of Managers have a plum—this is only a little one, it is true, but it *is* a plum—of the profession in their gift, that faithful service and attention to duty should not be remembered and rewarded.

### A PLEA FOR CHRONIC CASES.

"An ex-Private Nurse" writes: The aversion so many of our nurses show to the nursing of chronic cases is, I think, scarcely reasonable or creditable to our profession. One would not, of course, go to a home for incurables for training. It is right and desirable that every nurse should wish to see and nurse as many acute cases, and have as much practice as possible during her training, and be given every opportunity her hospital affords to perfect herself in knowledge and nursing skill. But when that knowledge and skill are gained, and the nurse has left hospital and taken up private nursing, it is surely unreasonable to expect that all her "cases" must be acute or "interesting" from a hospital point of view. The clergyman who has spent at least three years in preparation for his work does not expect to visit only dying parishioners, or wish to work only amongst thieves and murderers. The greater part of a solicitor's work is trivial, or at least routine; while, to come nearer home, we are well aware that the greater number of our most prosperous medical men have a large proportion of "cranks" on their lists. I remember a conversation which took place several years ago in my hearing between a general practitioner who had been away for a week and his assistant: "Mrs. —— tells me you have not visited her since Friday." "No, I have been rather busy, and you know there is really nothing the matter with her." "It does not matter to you whether she is ill or not; so long as she likes to pay me five shillings for a ten minutes' conversation about the weather she shall be visited." But to look at the subject from a higher point of view, what should we think of the family doctor if he refused to interest himself in the ordinary illnesses which flesh is heir to, the bronchitis, cardiac, and renal troubles, and the cases of cancer—generally very exacting patients—of whom he has often a dozen on his list at one time? But to each he brings—or tries to bring—the same fresh interest and cheerful spirit with which he would treat a rare or difficult case. And who shall say that he is less welcome, or less worthily occupied, when trying to prolong the life, or alleviate the sufferings, of such a patient than when fighting hand to hand with death in a case of enteric fever or pneumonia? Shall, then, we women, who are supposed to be so much more patient and pitiful than men, nurses who have deliberately chosen a life calling for all the more womanly virtues, turn away from those laid aside, and, soured, maybe, by unrealised dreams and ambitions not less high than our own? To many natures inaction is much harder to bear than pain. "Aye, I was strong and able-bodied, loved my work. But now I am a useless hulk; 'tis time I sank. I am in all men's way. I trouble them; I am a trouble to myself." "And I sit, and I am lonesome, and the times are few that any come to sit in my poor place, and talk awhile. Why should they come, forsooth?" We should all learn to take broader views of life and our duties. Private nursing is, in the majority of cases, not edifying; the family littlenesses, as well as the family skeletons, are often too closely seen, but surely the chronic cases are as much to be pitied as those who are ill only for a few weeks? In nineteen out of twenty cases trained nursing is of some use, more or less, and from the "crankiest" patients one derives, at least, a little harmless amusement. In addition, the nurses who despise the "nursery work" of children's wards or the uninteresting adult patients are not usually the best nurses for other work. Sympathy and thoroughness are most important qualifications in every branch of nursing, and these include the least, as well as the greatest, duties. "He that is faithful in little is faithful in much."

### THE QUESTION OF WEARING UNIFORMS INDOORS.

"Sister Ellen" writes: During the winter season I have been to many social functions, both public and private, in one of our large cities, and with rare exceptions has the hospital nurse been absent. I do not wish to prevent a nurse from enjoying her well-earned recreation, but I do object to her appearing in indoor uniform. I admit that most nurses look well in their caps and costumes, but I think the nursing dress unsuitable amongst others in evening attire. The wearing of it leads to many remarks not always of a complimentary nature. From another point of view, it is objectionable that people should be reminded of sickness, which the sight of a nurse suggests, when they are met together for social purposes. I do not consider that it is dignified for a nurse to wear her uniform, which is an emblem of her mission to the sick and dying, in theatres, concerts, and receptions, and have the eyes of everyone turned upon her. Some nurses say that they find uniforms an economy, but this I doubt, as so many additions are made to that allowed by most institutions. I have seen nurses appear with huge flowers arranged as sprays, which are most unbecoming with a cotton gown and apron. If matrons would be firm and forbid their nurses from wearing hospital uniform out of the wards, this display would disappear. When a nurse wishes to attend evening entertainment, why cannot she appear in quiet dress? I feel convinced that people are weary of seeing the nurse at every turn.

### NURSES' HOLIDAYS.

"A Leeds Nurse" writes: Do some members of the Bristol Board of Guardians want to make a lasting name for themselves? They are going the right way about it. "The evil that men do lives after them," &c. They have refused the title of sister to their nurses, and now want to decrease their holidays. Where there is any point of comparison between the lot of a tradesman's wife and a nurse, or, for the matter of that, any woman earning her living in the world, I am at a loss to understand. Their method of reasoning is contrary to all that of the most enlightened minds of the nineteenth century. The point of argument for supporting or opposing any measure is not whether it always has or has not been done, but whether it is "right" or "wrong." Unfortunately there is no settled standard of "right" and "wrong." Everyone fixes their own, and often enlarges or diminishes it to meet some particular case. It is certainly necessary for the health of a nurse that she should have more than three or four days' holiday, therefore "right." Doubtless many a tradesman's wife needs more, but because she cannot have it, is no reason why we should not. On the other hand, a tradesman's wife, and many other wives, can regulate their own homes and their work to a great extent, as health or fancy dictates, consequently have more chance of preserving their health and strength.

### Appointments.

Hospital for Women and Children, Edinburgh.—Miss C. M. Lyall has been appointed Lady Superintendent. She was trained for three years at St. Luke's Hospital, Halifax, where she was afterwards charge nurse of the obstetric and children's wards. She has been superintendent nurse of the East Parochial Hospital, Aberdeen, and has also done private work.

Rous Memorial Hospital, Newmarket.—Miss M. W. McDouall has been appointed Matron. She was trained at the London Hospital, and her last appointment was that of matron at the Royal Victoria Nursing Home, South Ascot.

Taunton and Somerset Hospital.—Miss Catherine S. Bulteel has been appointed Matron. She was trained at the London Hospital, and has since been night sister in the same institution.

The new Matron of Wolverhampton Hospital is Miss Eastmond, not Miss Eastwood.

### For Reading to the Sick.

### BENEATH THE CROSS.

Our pains are portioned to our powers,—His hand may hurt, but cannot harm;
But if the cross be on us laid, and our soul's crown of thorns be made,
Then, sure 'twere best to bear the cross, nor lightly fling the thorns behind,
Lest we grow happy, by the loss of what was noblest in the mind.
Here—in the ruins of my years—Master, I thank Thee through my tears;
Thou suffered'st here, and didst not fail; Thy bleeding feet these paths have trod;
But Thou wert strong, and I am frail; and I am man, and Thou art God!
How I have striven, Thou know'st! Forgive how I have failed, who saw'st me strive! —*Lytton.*

There is no grief that ever wasted man
But finds its hour here in Thine awful week.
—*Keble.*

The wine of Love can be obtained of none,
Save Him who trod the wine-press all alone.
—*Trench.*

**Reading.**

We are called to watch by the Cross, to listen to, and to learn new lessons from those sacred and well-known words, in which our dying Redeemer broke the silence of the dead and dark hours of the first Good Friday. . . . We can never exhaust that most stupendous subject, the passion of Jesus. It is the climax of the perfect life. It is the culminating struggle between good and evil. It is the fathomless measure of the love for each single human soul that beats in the very heart of God. It is our hope and strength in life, our stay in death, our passport to paradise.

The seven words from the Cross! We come back to them year after year; we find them always new; we find that they have always fresh lessons to teach us; for He who spoke them is our Lord and our Redeemer, who measured life and death at their true value, and who upon the cross shows us the value of every human soul as it appears stripped and under the eye of God, even as He hung stripped and naked, during those dark hours. . . . If we were commemorating merely the death of one who died, then Good Friday would be without its inspiration of life and of hope. But triumph, victory, is the keynote of it all. And He by whose cross we would watch says, "Be brave-hearted, be men of a great courage. Be of good cheer, I have overcome the world."

Only the sad story shows us how the victory was won; only it shows me what my sin has done for my best, my truest, my dearest Friend; only it shows me what Jesus meant when he said, "Come, take up thy cross and follow me."—*Isaacs.*

Up Thy Hill of Sorrows,
Thou all alone;
Jesus, man's Redeemer,
Climbing to a Throne.
Thro' the world triumphant,
Thro' the Church in pain,
Which think to look upon Thee
No more again.

Upon Thy Hill of Sorrows
I, Lord with Thee;
Cheered, upheld, yea, carried,
If a need should be.
Cheered, upheld, yea, carried,
Never left alone;
Carried in Thy heart of hearts
To a Throne.

—*Christina Rossetti.*

### Notes and Queries.

THE Editor is always willing to answer in this column, without any fee, all reasonable questions, as soon as possible.

But the following rules must be carefully observed :—

1. Every communication must be accompanied by the name and address of the writer.
2. The question must always bear upon nursing, directly or indirectly.

If an answer is required by let'er a fee of half-a-crown must be enclosed with the note containing the inquiry.

*Baths.*

(1) Will you kindly tell me which is the nearest town to Warwick or to St. Leonards on Sea, at which I can obtain electric baths for rheumatic gout ?—*Beatrice.*

You could probably find out by making inquiries on the spot that you could obtain the baths in St. Leonards, as these are now provided by many private homes and hydropathic establishments all over the country. Also it might be worth while making inquiries at Leamington, as it is distinctly a health resort.

*Pictures.*

(2) Can you tell me the name of any society which would give pictures to a deserving hospital ? Would an application to the Kyrle Society be any use ? If so, can you give me the address of that society ?—*B. C.*

The address of the Kyrle is 49, Manchester Street, W. By making your request known through the local press you would probably receive a satisfactory response from the wealthier residents in the neighbourhood.

*Home for the Aged.*

(3) Will you kindly tell me if there is a home for aged and infirm men anywhere ? I am asking for a friend, who would be willing to pay a small sum weekly, though her means are limited, on condition that her father were well looked after, as she is a teacher, and unable to see to him herself.—*Nurse.*

It is exceedingly difficult to find cheap homes for the aged. Those cared for by the Little Sisters of the Poor (Portobello Road, W.) and the Sisters of Nazareth (Nazareth House, Hammersmith) are most numerous and widely distributed.

*Monthly Nursing.*

(4) Will you please tell me where I can get a good training in monthly nursing at the least possible cost ?—*E. L. B.*

Good and cheap training in monthly nursing is offered by the British Lying-in Hospital, Endell Street, W.C. (£7 3s.); the City of London Lying-in Hospital, City Road, E.C. (£7 7s.); and the East End Mothers' Home, Commercial Road, E., &c.

*Dictionary.*

(5) Is there a better medical dictionary than "Hoblyn's ?"—*Enquirer.*

There are many medical dictionaries to suit different readers. "Gould's" or "Lippincott's" are much larger than "Hoblyn's," but a pocket edition of each is published.

*Probationer.*

(6) Will you kindly tell me (1) if there is any hospital or training home where they take young ladies of 25 as probationers : and (2) if a first-aid certificate (St. John Ambulance) would be any recommendation ?—*M. M. M.*

(7) Would you kindly let me know of any hospital or infirmary where I would be accepted as probationer free, and where a small salary would be given ? I am 25 years of age.—*S. A. C.*

1. Twenty-five is a most acceptable age with almost all hospital authorities. Apply to the matrons of suitable institutions. 2. No. Previous training is a disadvantage. See the "Nursing Profession : How and Where to Train" for information.

*Birmingham.*

(8) Will you kindly forward to me a list of nursing homes at Birmingham, and say if they are worked on the co-operative system ?—*J. J. R.*

The nursing associations managed by committees in Birmingham are the Birmingham and Midland Counties Training Institution for Nurses, 12, The Crescent ; the Birmingham District Nursing Society, 41, Waterloo Street, Birmingham ; and the Queen's Hospital External Nursing Department, Bath Row. None are upon the co-operative system.

*Paris.*

(9) Two retired nurses, wishing to add to their income, think of taking a house in Paris, where English people, who may be taken ill during their visit to the Exhibition, could be received and nursed by their own countrywomen. What would the Editor advise ?—*M. A. P.*

We cannot advise. We can but point out that although it is possible that there may be an opening for an English nursing home in Paris at Exhibition time, all such ventures are attended with a considerable amount of risk. If you have a large circle of acquaintances likely to go to Paris this year they might be glad to turn to you in case of need ; but a temporary venture like the one proposed would require extensive advertising, which would, we fear, eat up the profits.

*Swiss Hospitals.*

(10) Would you kindly let me know how to get into a Swiss hospital after a recognised course of training in England, and if there are likely to be vacancies in such hospitals ?—*A. L. S.*

We have no official returns of the nursing in Swiss hospitals. Perhaps one of our readers would kindly answer this query from personal experience.

*Defective Morality.*

(11) May I ask you kindly to assist me in finding a home for a young woman of defective morality ? Dr. Oldfield, after carefully examining her, says it is evidently a case of defective mental development, but assures me that under wise control she might become a useful member of society. She is sturdy and strong, capable of manual labour. He says she should be fed on bread and water and fruit, with plenty of green vegetables, such as watercress, &c., and advises me to get her a situation as servant with a strong-minded mistress, preferably a nurse, or one accustomed to mental cases, and where there are no young men in the home, offering to pay a small sum for board.—*E. M. O.*

Can any of our readers help "E. M. O." ?

*Sunday Visiting.*

(12) Could you tell me how to proceed in order to gain admittance to a hospital on Sundays for the purpose of chatting to and in any way helping the patients ?—*P. G. C.*

Write to the secretary of the institution you propose visiting and ask what arrangements are in force for Sunday visitors.

*Training.*

(13) Having had an offer to enter a children's hospital for training as paying probationer, I should feel greatly obliged if you would let me know if the advantages are as great as those of being trained in an adults' hospital—I mean after the certificate has been obtained ?—*L. H.*

No. You must hold a three years' certificate from a general hospital in order to be on an equal footing with other nurses.

*Aged Convalescent.*

(14) Can you kindly inform me of any convalescent home where the limit to age would not preclude a man of 77 from being received ?—*N. H.*

Most convalescent homes are willing to receive aged cases if they are not helpless and do not require an undue amount of attention.

*Home for Infant.*

(15) Could you tell me of a home or public institution where a baby o three months could be received, permanently, with small weekly payment ?—*M. P.*

Dr. Barnardo has a home for babies. Write for particulars to the Offices, 18-26, Stepney Causeway, E.

*Dandruff.*

(16) Will you kindly tell me if you know of a cure for dandruff ? 2. Can you tell me if it is the cause of the hair turning prematurely grey ?—*E. C.*

See the article by Leslie Phillips, M.D., in THE HOSPITAL for July 31st, 1879. Also refer to answer headed "Dandruff and Constipation" in "Notes and Queries" of November 4th, 1899. I should advise you consulting a doctor if simple remedies fail.

*Epileptics.*

(17) May I ask if you can tell me whether there is a home for epileptics where they will take as a servant a woman who is subject to slight fits but who is quite able to earn her own living, though not fit for service in a private family ? She is a thoroughly good servant, and can cook very well.—*M. C.*

It might be worth while writing the Secretary, the Hospital for the Paralysed and Epileptic, Queen's Square, Bloomsbury ; and the Secretary, National Society for the Employment of Epileptics, 12, Buckingham Street, Strand.

*Lip Reading.*

(18) Can you tell me of any institution (easily reached from Walton-on-Thames) where instruction in lip reading could be obtained ?—*E. A. R.*

The Director, Training College for Teachers of the Deaf and Dumb, 11, Fitzroy Square, W., could probably recommend one.

*Madeira.*

(19) Is there any British hospital or nursing institute in Madeira where a nurse with one year's certificate could get employment for the winter ? Where ought I to apply for particulars ?—*Nurse Constance.*

Apply to the Secretary, the Colonial Nursing Association, the Imperial Institute, S.W., for information. Your training is too short for the requirement's of the Association's staff.

*Standard Books of Reference.*

"The Nursing Profession : How and Where to Train." 2s. net.
"The Nurses' Dictionary of Medical Terms." 2s. 6d. net.
"Burdett's Series of Nursing Text-Books." 1s. each.
"A Handbook for Nurses." (Illustrated.) 5s.
"Nursing : Its Theory and Practice." New Edition. 3s. 6d.
"Helps in Sickness and to Health." Fifteenth Thousand. 5s.

All these are published by THE SCIENTIFIC PRESS, Ltd., and may be obtained through any bookseller or direct from the publishers, 28 & 29, Southampton Street, London, W.C.

## Figures and Tables

**Figure f1:**
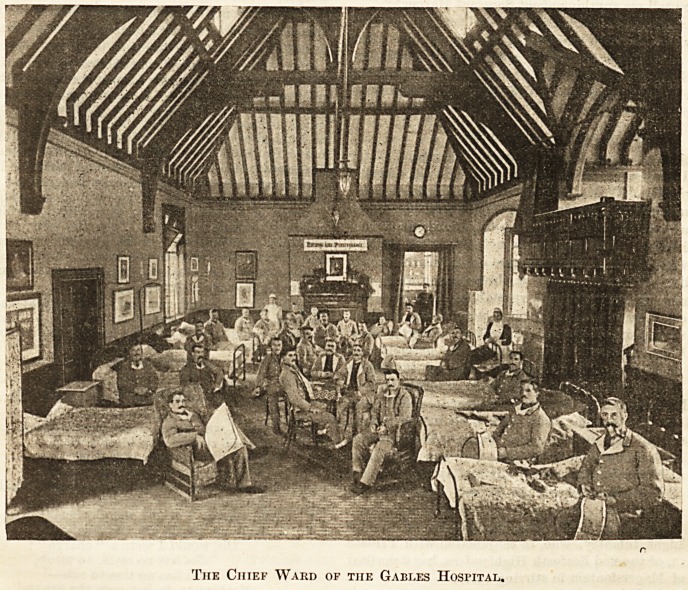


**Figure f2:**
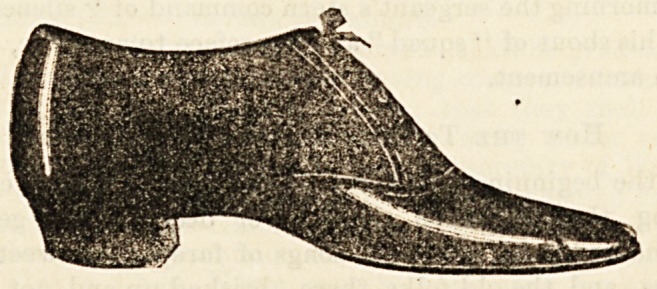


**Figure f3:**